# Htt is a repressor of Abl activity required for APP-induced axonal growth

**DOI:** 10.1371/journal.pgen.1009287

**Published:** 2021-01-19

**Authors:** Claire Marquilly, Germain U. Busto, Brittany S. Leger, Ana Boulanger, Edward Giniger, James A. Walker, Lee G. Fradkin, Jean-Maurice Dura

**Affiliations:** 1 IGH, Centre National de la Recherche Scientifique, Univ Montpellier, Montpellier, France; 2 Center for Genomic Medicine, Massachusetts General Hospital, Boston, Massachusetts, United States of America; 3 Intramural Research Program, NINDS, NIH, Bethesda, Maryland, United States of America; 4 Department of Neurology, Massachusetts General Hospital, Harvard Medical School, Boston, Massachusetts, United States of America; 5 Cancer Program, Broad Institute of MIT and Harvard, Cambridge, Massachusetts, United States of America; 6 Department of Neurobiology, University of Massachusetts Medical School, Worcester, Massachusetts, United States of America; Duke-NUS Medical School, SINGAPORE

## Abstract

Huntington’s disease is a progressive autosomal dominant neurodegenerative disorder caused by the expansion of a polyglutamine tract at the N-terminus of a large cytoplasmic protein. The *Drosophila huntingtin* (*htt*) gene is widely expressed during all developmental stages from embryos to adults. However, *Drosophila htt* mutant individuals are viable with no obvious developmental defects. We asked if such defects could be detected in *htt* mutants in a background that had been genetically sensitized to reveal cryptic developmental functions. Amyloid precursor protein (APP) is linked to Alzheimer’s disease. Appl is the *Drosophila* APP ortholog and *Appl* signaling modulates axon outgrowth in the mushroom bodies (MBs), the learning and memory center in the fly, in part by recruiting Abl tyrosine kinase. Here, we find that *htt* mutations suppress axon outgrowth defects of αβ neurons in *Appl* mutant MB by derepressing the activity of Abl. We show that *Abl* is required in MB αβ neurons for their axon outgrowth. Importantly, both *Abl* overexpression and lack of expression produce similar phenotypes in the MBs, indicating the necessity of tightly regulating Abl activity. We find that Htt behaves genetically as a repressor of Abl activity, and consistent with this, *in vivo* FRET-based measurements reveal a significant increase in Abl kinase activity in the MBs when Htt levels are reduced. Thus, Appl and Htt have essential but opposing roles in MB development, promoting and suppressing Abl kinase activity, respectively, to maintain the appropriate intermediate level necessary for axon growth.

## Author summary

Understanding the normal physiological roles of proteins involved in neurodegenerative diseases can provide significant insight into disease mechanisms. *Drosophila* offers a powerful system in which to ask these fundamental questions. Both Htt, related to Huntington’s disease, and Appl, related to Alzheimer’s disease, have well-conserved single orthologs in the fly genome. Appl has been shown to be a conserved modulator of a Wnt-PCP signaling pathway required for axon outgrowth in the mushroom body (MB) in the *Drosophila* brain. However, roles for Htt in fly brain development have not been reported. Unexpectedly, we found that *htt* mutations suppress the axon outgrowth defects of *Appl* mutants in the MB, indicating a link between these two neurodegenerative proteins and a cryptic role of Htt during development. Abl tyrosine kinase is a downstream effector of the Appl receptor, and we show here that Abl is also required for MB axon outgrowth. Importantly, Abl activity must be tightly regulated as evidenced by our observations that both under and overexpression of Abl result in similar axonal defects. We demonstrate that Htt is an inhibitor of Abl activity and provide evidence that the phenotypic rescue of αβ axons in *Appl* mutants by reducing *htt* is mediated by the restoration of proper levels of Abl signaling. These data, therefore, suggest that Appl and Htt act antagonistically to maintain an optimal balance of activation and inhibition of Abl, and thereby promote the growth of MB αβ axons.

## Introduction

Neurodegenerative disease (ND) encompasses a large and heterogeneous group of maladies, including many that are associated with accumulation of specific misfolded proteins [1]. Despite their variety, however, these diseases share a number of cellular pathologies [2,3], raising the question of whether different ND-associated genes might function in shared genetic pathways. Several of these disease genes, moreover, have been implicated in neurodevelopmental processes [4–8], suggesting that studies of development may be an effective strategy to reveal initially cryptic connections among genes implicated in ND.

Huntington’s disease (HD) is a progressive, autosomal dominant, neurodegenerative disorder. It is a monogenic disease caused by the expansion of a polyglutamine (polyQ) tract at the N-terminus of a large cytoplasmic protein (3144 a.a.), huntingtin (Htt) [9]. Several studies indicate that an alteration of wild-type Htt function might also contribute to disease progression [4]. Consistent with this, numerous biochemical and *in vitro* studies have suggested that Htt functions in mammalian neuronal development, synaptic function and axonal trafficking [9]. While HD has been characterized as a neurodegenerative disease, a recent study indicates it is also required for normal human brain development [8].

*Drosophila* has been useful previously as a model to examine the effects of polyQ-expanded human huntingtin transgenes on neuronal form and function [10,11]. The fly huntingtin protein (Htt, 3583 a.a.), although lacking a polyQ tract, is similar to the human Htt protein, with four regions of high sequence homology clustered along the protein in the N-, central- and C-terminal regions. Fly Htt is expressed ubiquitously at low level in embryos, larval and adult tissues, with no specific pattern of expression. Fly Htt is found predominantly in the cytoplasm, even when overexpressed [12]. Despite *htt* being highly conserved across all *Drosophila* species, indicating an essential role for biological fitness, null *htt* mutants display no gross developmental defects [12,13], although brains from *Drosophila* Htt mutants have reduced axon complexity [12]. This suggests that it could be necessary to alter the expression of another gene (or genes) during development to be able to detect a *htt* loss of function phenotype. Therefore, we asked whether mutant *htt* modifies a brain axon growth defect present when another neurodegeneration-related protein is lacking, i.e., in a sensitized genetic background.

In contrast to HD, Alzheimer’s Disease (AD) is highly genetically complex [14–16]. Like HD, AD is also viewed as a proteinopathy, since it is associated with accumulation of amyloid fibrils derived from the Amyloid Precursor Protein (APP). APPs have therefore been investigated intensely, however their normal function in the brain remains unclear and controversial. *Drosophila* encodes a single APP homologue, called *Appl*, that is expressed in all neurons throughout development. It has been shown that *Appl* is a conserved neuronal modulator of a Wnt planar cell polarity (Wnt/PCP) pathway [5]. This signaling pathway is essential for proper axon outgrowth in the learning and memory center of the fly, a bilaterally symmetric pair of structures called the Mushroom Bodies (MB). In this context, it has been proposed that Appl is part of the membrane complex formed by the core PCP receptors, and further that it promotes phosphorylation of the Dishevelled (Dsh) cytoplasmic adaptor protein. Dsh is a core component required for all known Wnt pathways, including the Wnt/PCP pathway [17,18]. Specifically, Appl recruits a non-receptor protein tyrosine kinase, called Abl, to the PCP receptor complex and positively modulates its phosphorylation of Dsh [19]. Consistent with this view, it has been shown that a 50% reduction of *Abl* leads to enhancement of the *Appl* mutant phenotype. Conversely, overexpression of wild-type *Abl*^+^, but not a kinase-dead version, in the MB neurons led to a strong rescue of the *Appl* mutant phenotype. Together with accompanying biochemical experiments, these data suggested that Appl promotes the phosphorylation of Dsh by Abl kinase, and further showed that this mechanism is conserved in mammals [5]. Thus, Abl is a key downstream effector of Appl required for it to stimulate MB axon outgrowth.

The Abl family of non-receptor tyrosine kinases includes human ABL1 and ABL2 as well as *Drosophila Abl*. Each Abl protein shares a conserved domain structure consisting of a SH3-SH2-TK (Src homology 3-Src homology 2-tyrosine kinase) domain cassette which confers autoregulated kinase activity. A carboxy-terminal F (F-actin-binding) domain ties Abl-dependent phosphoregulation to actin filament reorganization [20]. ABL1 has been implicated in a range of cellular processes including actin dynamics and cell migration. Abl was discovered as a cellular proto-oncogene that is constitutively active in human chronic myelogenous leukemia and acute lymphocytic leukemia [21]. Kinase activity of Abl *in vivo* is limited both by intramolecular interactions [22] and by cellular inhibitors, such as Pag/Msp23 [23]. After removal of inhibition, Abl acquires substantial catalytic activity that is further enhanced by primary and secondary (auto)phosphorylation [24]. The Abl kinases have also been shown to play a crucial role in the development of the nervous system. Overexpression of active Abl in adult mouse neurons results in neurodegeneration and neuroinflammation and activation of Abl has been shown to occur in human neurodegenerative disease [25]. In contrast to mammalian Abl, *Drosophila* Abl has not been shown to directly cause tissue hyperplasia or cell fate transformation *in vivo*, but rather is essential for cell adhesion and morphogenetic processes such as axonogenesis and growth cone motility [26–28]. Several studies have shown that the precise level of Abl activity is critical to its axonal function, with loss- and gain-of-function both leading to severe defects in neural patterning [29–31]. Recent experiments revealed the cell biological and biophysical basis of this relationship, showing that either increased or decreased levels of Abl activity induce disorder in growth cone actin and thereby greatly augment the frequency of stochastic errors in growth and guidance [32,33]. Of particular relevance here, *Abl* has been implicated in axonal arborization and growth in the *Drosophila* brain [34], including the MBs [5], though the precise role of *Abl* in normal MB development has not been documented.

The MBs, together with the central complex, form the core of the adult central brain of *Drosophila*. Due to extensive study, they offer an exceptionally powerful system for analyses of genetic and molecular mechanisms of development and function. The MBs are two bilaterally symmetric structures that are required for learning and memory [35,36]. Each MB is comprised of 2000 neurons that arise from 4 identified neuroblasts. Three types of neurons appear sequentially during development: the embryonic/early larval γ, the larval α’β’ and the late larval/pupal αβ. Each αβ neuron projects an axon that branches to send an α branch dorsally, which contributes to the formation of the α lobe, and a β branch medially, which contributes to the formation of the β lobe [37]. Both lobes require the PCP mechanism for efficient axon extension [38]. The PCP genes, however, do not act cell-autonomously to promote MB axon growth. Rather, PCP produces a “community effect” that coordinates the growth decisions of large groups of MB axons, and that overrides the effect of mutations in single PCP components in any single axon or cluster of axons. [38]. Appl is required for this PCP-dependent ‘community effect’. In β-branches, Appl is evidently also required for some other mechanism that acts cell-autonomously, in addition to its contribution to the non-cell-autonomous PCP mechanism [5]. The molecular nature of this second autonomous Appl function remains unknown.

Here we investigate the genetic and functional interactions of *Htt*, *Appl*, *Abl* and the core PCP gene *dsh* in *Drosophila*. We find that *htt* mutations suppress the MB axonal outgrowth defects observed in *Appl* mutants. Since Abl is known to act downstream of Appl it seemed a potential target for *htt* mutant-induced suppression of the *Appl* phenotype. We therefore next characterized the role of Abl in normal MB development. Using analysis of *Abl* loss-of function (LOF) alleles in single-cell MARCM clones we show that *Abl* is required for axonal growth in the developing αβ neurons of the MBs and that it is expressed in these neurons. Importantly, the overexpression of *Abl* in these neurons also leads to axonal growth defects, suggesting the possible existence of cellular proteins that negatively control neuronal Abl activity. Finally, we demonstrate that Htt acts as a cellular inhibitor of Abl activity, both genetically, as it functions antagonistically to Abl in MB axon growth, and biochemically, as FRET measurement of Abl kinase activity *in vivo* reveals that is derepressed by reducing Htt expression. These results indicate that *Appl* and *htt*, whose human homologs are central players in neurodegeneration, regulate Abl kinase in opposite directions to maintain its activity in the narrow range necessary for normal axon outgrowth in MB αβ neurons.

## Results

### Reduction of *htt* rescues the MB β axon outgrowth phenotypes of *Appl* mutants

**Fig 1:**
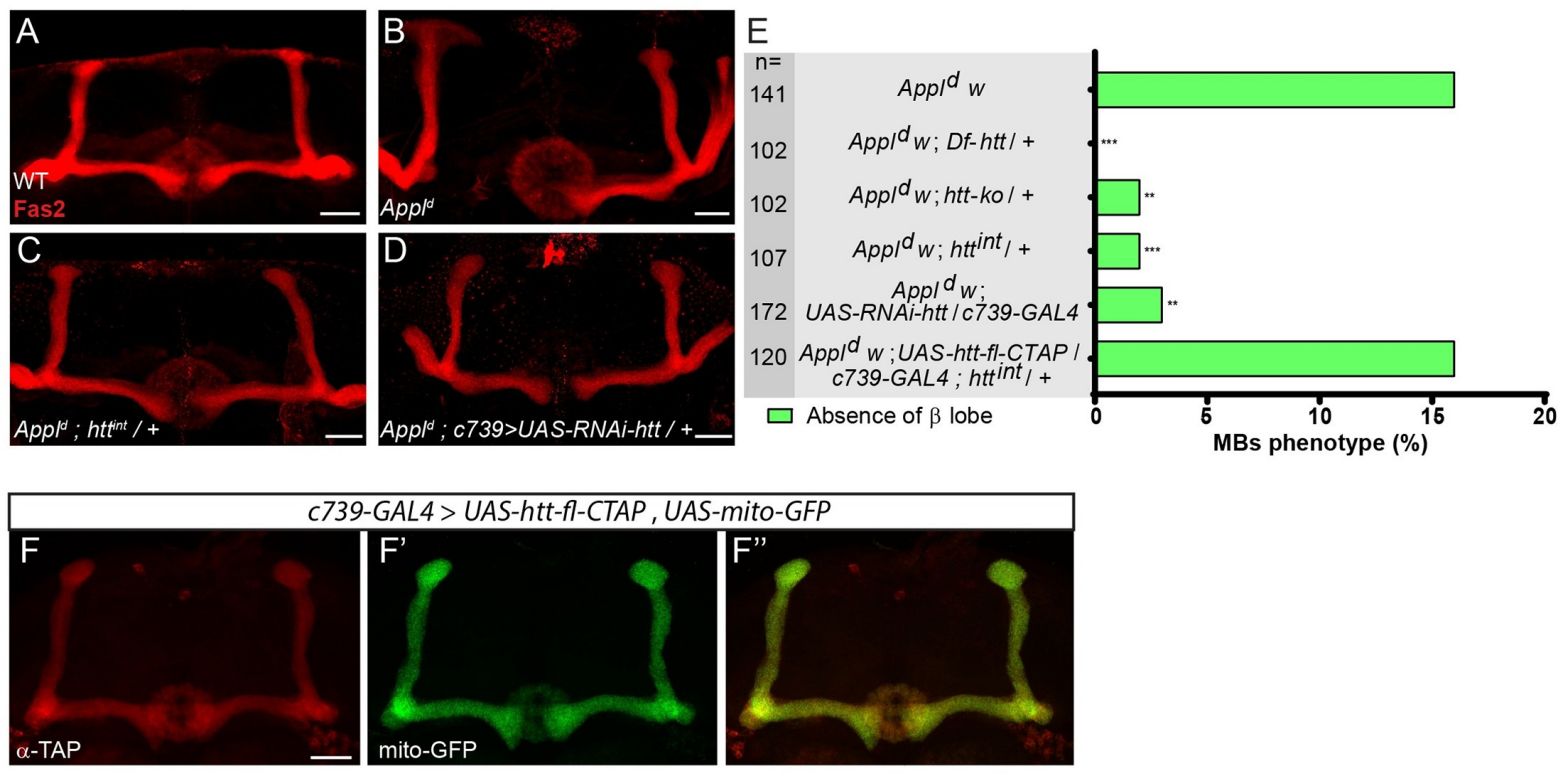
The loss of *htt* rescues the *Appl*^*d*^ MB axon outgrowth mutant phenotype. (A) Wild-type MB α and β lobes revealed by anti-Fas2 staining. (B-D) Anti-Fas2 staining reveals the absence of the β lobe in an *Appl*^*d*^ mutant brain (B), which is rescued by the loss of one copy of *htt* (C) and is also rescued by the expression of RNAi against *htt* driven by *c739-GAL4* (D). (E) Quantitation of rescue of the MB *Appl*^*d*^ phenotype by *Df-htt*, *htt-ko*, *htt*^*int*^ and *UAS-RNAi-htt* driven by *c739-GAL4*. The rescue of *Appl*^*d*^ by *htt*^*int*^ is prevented by the overexpression of *htt* driven by *c739-GAL4* indicating the functionality of the *UAS-htt-fl-CTAP* transgene. n = number of MBs analyzed, ** P < 0.01 and *** P < 0.001. Significance was calculated by a multiple comparison Fisher’s exact test (P = 5.8 10^−10^) followed by post-hoc Bonferroni’s multiple comparison correction (p-values = 2.3 10^−5^, 0.0018, 0.0009, 0.0014 and 1). (F-F”) The expression of *UAS-htt-fl-CTAP* revealed by an anti-TAP staining (F) and *UAS-mito-GFP* (F’) driven by *c739-GAL4* are similar in the MBs (F”). All panels correspond to adult brains. The scale bar on panels A-D and F indicates 30 μm. Images are composite stacks to allow the visualization of axon trajectories along their entire length. Full genotypes: (A) *y w*^*67c23*^ / Y; *c739-GAL4 UAS-mito-GFP / +*. (B) *Appl*^*d*^ w*/ Y*; c739-GAL4 UAS-mito-GFP / +*. (C) *Appl*^*d*^ w* / Y*; c739-GAL4 UAS-mito-GFP /+; htt*^*int*^ */ +*. (D) *Appl*^*d*^ w* / Y; *c739-GAL4 UAS-mito-GFP / UAS-RNAi-htt*. (E) top to bottom: *Appl*^*d*^ w* / Y*; c739-GAL4 UAS-mito-GFP / +*. *Appl*^*d*^ w* / Y*; c739-GAL4 UAS-mito-GFP / +; Df-htt / +*. *Appl*^*d*^ w* / Y*; c739-GAL4 UAS-mito-GFP / +; htt-ko / +*. *Appl*^*d*^ w* / Y*; c739-GAL4 UAS-mito-GFP / +; htt*^*int*^ */* +. *Appl*^*d*^ w* / Y*; c739-GAL4 UAS-mito-GFP / UAS-RNAi-htt*. *Appl*^*d*^ w* / Y*; c739-GAL4 UAS-mito-GFP / UAS-htt-fl-CTAP*; *htt*^*int*^ / +. (F-F”) *y w*^*67c23*^ / Y*; c739-GAL4 UAS-mito-GFP / UAS-htt-fl-CTAP*.

*Appl* null (*Appl*^*d*^) flies are viable, fertile and display no gross structural defects in the brain [39]. However, *Appl*^*d*^ MBs display modestly-penetrant axonal defects in αβ neurons [5]. For simplicity, in our quantification of this phenotype we will focus our attention on the role of *Appl* in β-branches, where *Appl* is required cell-autonomously [5]. From 3110 *Appl*^*d*^ MBs, 451 (14.5%) showed an absence of β lobe phenotype (see details in Material and methods), in accordance with what was previously reported [5]. Two control MBs (0.26%) displayed β lobe absence out of 759 MBs (see details in Material and methods). In line with our interest in potential developmental associations of neurodegenerative genes, we wondered whether *htt* would modify the effect of *Appl* in MB αβ neurons. By itself, as expected from published data [12] the loss of one or even two copies of *htt* did not result in any significant MB developmental defects (S1A Fig). However, we found that mutation of *htt* potently suppresses the MB *Appl*^*d*^ mutant phenotype in αβ neurons (Fig 1). Specifically, the *Appl*^*d*^ MB phenotype was rescued by reducing *htt* expression using three different genetic manipulations: 1) *htt* heterozygosity using two mutant alleles of *htt* (*htt-ko* or *htt*^*int*^), 2) heterozygosity for a 55 kb chromosomal deficiency uncovering the *htt* locus and most of the adjacent *CG9990*, and 3) *htt* RNAi knock down in the αβ MB neurons (Fig 1A–1E; RNAi expression was driven in this experiment by the αβ neuron-specific *GAL4* line *c739*). Conversely, when *htt* was overexpressed in the MB αβ neurons using *UAS-htt-fl-CTAP*, a UAS-C-terminally TAP-tagged full length *htt*, it did not rescue *htt*^*int*^/+ (16% absence of β lobe in *Appl*^*d*^; *UAS-htt-fl-CTAP/c739-GAL4; htt*^*int*^/+ vs 2% in *Appl*^*d*^; +/+*; htt*^*int*^/+ and 16% in *Appl*^*d*^) (Fig 1E). As expected from the above genetic results, htt-fl*-CTAP* protein, like wild type Htt, was present throughout the axon when expressed in αβ neurons (Fig 1F).

**Fig 2:**
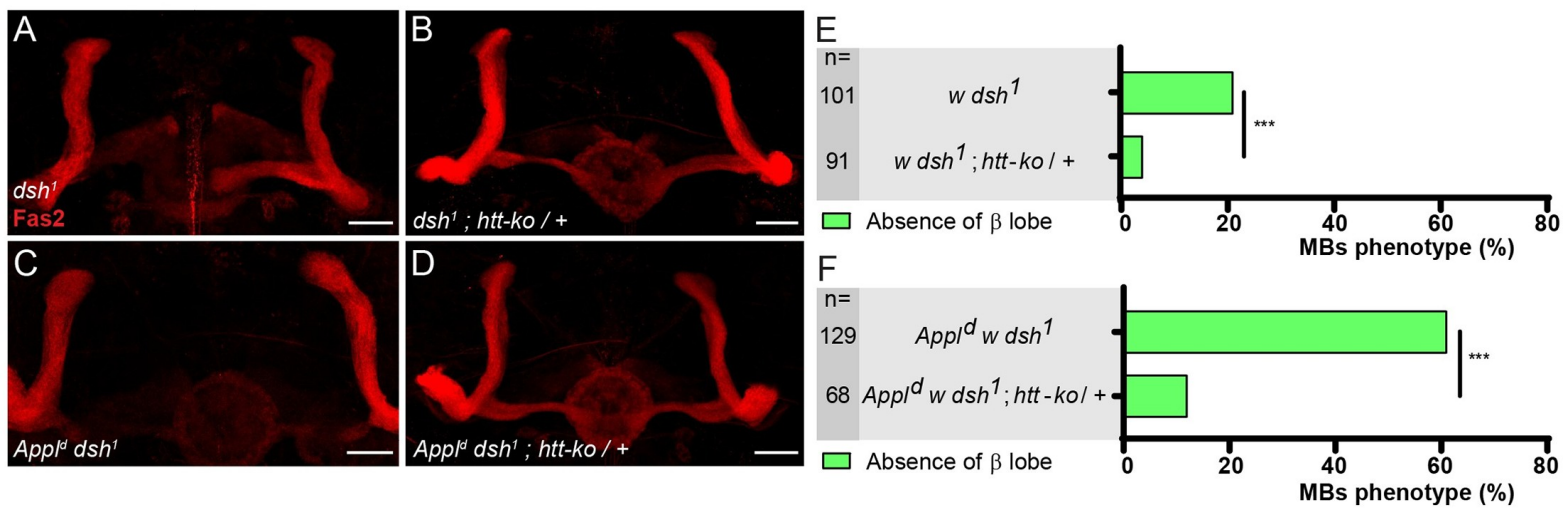
The loss of *htt* rescues the *dsh*^*1*^ MB axon outgrowth mutant phenotype. (A-B) Anti-Fas2 staining reveals the absence of β lobe in a *dsh*^*1*^ mutant brain (A), which is rescued by the loss of one copy of *htt* (B). (C-D) Anti-Fas2 staining reveals the loss of the two β lobes in an *Appl*^*d*^ *dsh*^*1*^ mutant brain (C), which is rescued by the loss of one copy of *htt* (D). (E-F) Quantitation of the rescue of *dsh*^*1*^ (E) and of *Appl*^*d*^ *dsh*^*1*^ (F) phenotypes by *htt-ko*. n = number of MBs analyzed and *** P < 0.001 (Fisher exact test). All panels correspond to adult brains. The scale bar on panels A-D indicates 30 μm. Images are composite stacks to allow the visualization of axon trajectories along their entire length. Full genotypes: (A) *w dsh*^*1*^. (B) *w dsh*^*1*^*;; htt-ko / +*. (C) *Appl*^*d*^ w* dsh^*1*^. (D) *Appl*^*d*^ w* dsh^*1*^*;; htt-ko / +*. (E) top to bottom: *w dsh*^*1*^. *w dsh*^*1*^*;; htt-ko / +*. *Appl*^*d*^ w* dsh^*1*^. *Appl*^*d*^ w* dsh^*1*^*;; htt-ko / +*.

Since Dsh is a core intracellular component of the Appl-dependent Wnt/PCP pathway in the MBs, we tested the potential interactions between *htt* and *dsh*. We found that removing one copy of *htt* also suppressed the *dsh*^*1*^ MB phenotype in the β-lobe (21% absence of β lobe in *dsh*^*1*^; *+/+* vs 4% in *dsh*^*1*^; *htt-ko /+—*Fig 2A, 2B and 2E upper bars). Moreover, hemizygosity for both *Appl* and *dsh* resulted in a strong MB axon outgrowth phenotype (*Appl*^*d*^ *w dsh*^*1*^*;* 60% absence of β lobe, vs 21% in *dsh*^*1*^ and 16% in *Appl*^*d*^). We found that heterozygosity for *htt* also strongly suppressed the *Appl*^*d*^ *dsh*^*1*^ phenotype (12% of absence of β lobe—Fig 2C–2E lower bars). Taken together, these data strongly suggest that Htt is a negative regulator of the Appl-dependent Wnt-PCP signaling pathway acting during MB β-axon outgrowth.

### *Abl* loss-of-function and gain-of-function mutants induce similar MB αβ neuron phenotypes

Since Abl is a key component of the Appl-dependent Wnt-PCP signaling pathway required for axon growth in MBs [5] and Abl was previously shown to phosphorylate Dsh [19], we sought to clarify the potential relationship between Abl, Appl, and Dsh in MB αβ neurons. Indeed, we found that the axonal phenotypes of *Appl*^*d*^ and *dsh*^*1*^ MB αβ neurons can be rescued by modest enhancement of Abl expression, through transgenic expression of an *Abl-GFP* fusion under control of *Abl* upstream genomic sequences (S1B and S1C Fig). We therefore characterized in more detail the axonal morphology phenotype in *Abl* loss- and gain-of-function MB αβ neurons.

**Fig 3:**
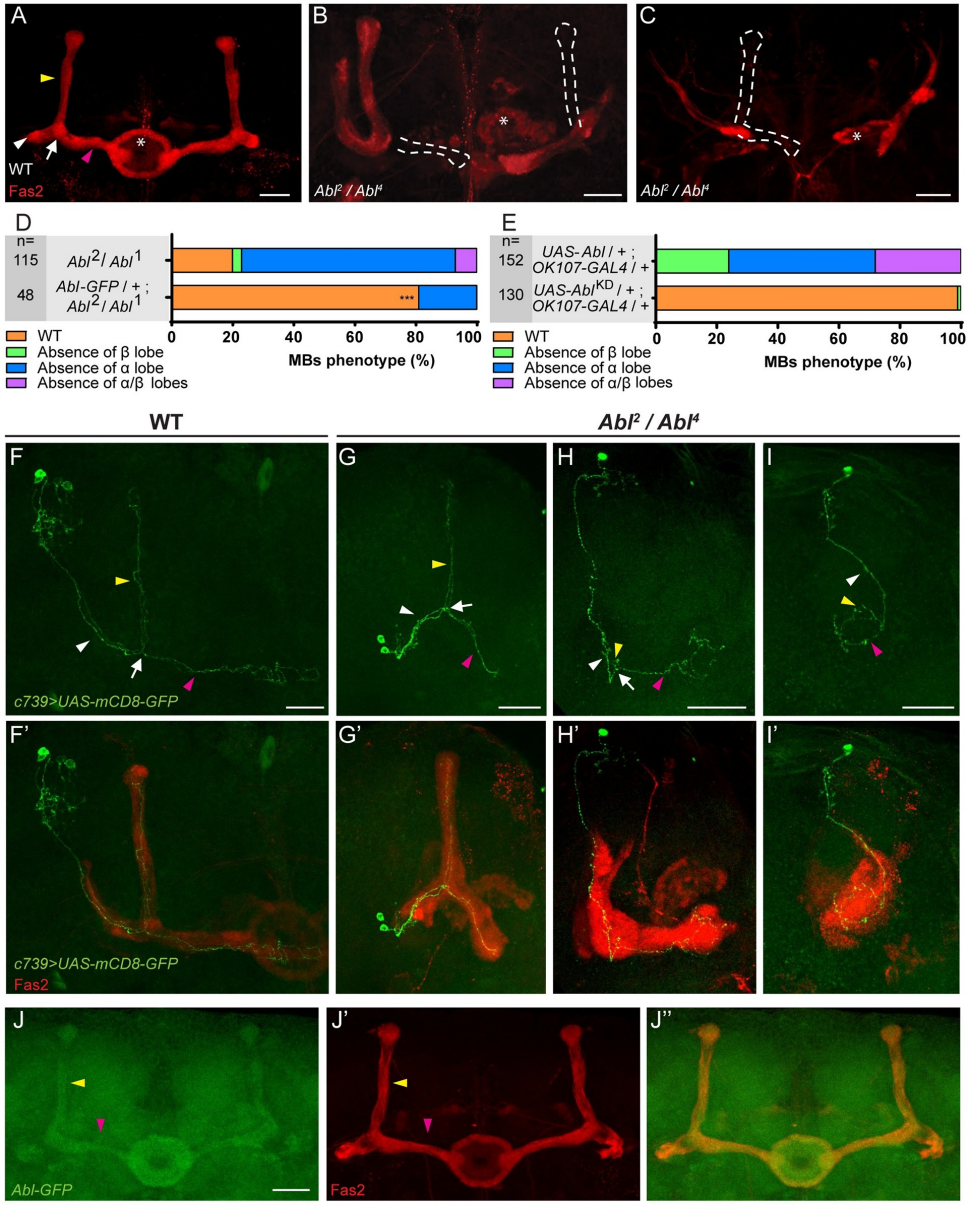
Either loss or overexpression of *Abl* affect MB αβ neuron morphology. (A-C) Anti-Fas2 staining on wild-type (WT) brain (A) and on *Abl*^*2*^*/Abl*^*4*^ brain (B-C). In a wild-type (WT) brain, the α lobe (indicated by yellow arrowhead) projects vertically and the β lobe, indicated by pink arrowhead, projects toward the midline and stops before reaching it. The loss of the β and α lobes (B) and of both the α and β lobes (C) is emphasized by white dashed lines. * shows the ellipsoid body. (D) Quantitation of the αβ neuron mutant phenotype in the *Abl*^*2*^*/Abl*^*1*^ mutant and rescued brains with the *Abl-GFP* genomic transgene. n = number of MB observed and *** P < 0.001 (Fisher exact test). Transgenic expression of *Abl-GFP* rescued the morphological defect and lethality of the double heterozygous *Abl*^*2*^/*Abl*^*1*^ combination, but failed to rescue *Abl*^*2*^ homozygote lethality, indicating that this lethality is due to associated modifiers on the chromosome independent of *Abl*. (E) Quantitation of the αβ neuron mutant phenotype when a wild-type or a kinase dead form of *Abl* expression is driven in the MBs by *OK107-GAL4* (n = number of MB observed). (F-F’) Two-cell WT αβ neuron MARCM clone in a WT brain (F) associated with anti-Fas2 staining in red (F’). (G-G’) Two-cell WT-looking αβ neuron clone (G) associated with anti- Fas2 staining in red (G’) in an *Abl*^*2*^*/Abl*^*4*^ brain. (H-H’) A single-cell αβ neuron clone with an α branch growth defect (H) associated with anti-Fas2 staining in red (H’) in an *Abl*^*2*^*/Abl*^*4*^ brain displaying an absence of α lobe. Note the α branch which stops just after the branching point in H (yellow arrowhead). (I-I*’*) A single-cell αβ neuron clone with α and β branch growth defects (I) associated with anti-Fas2 staining in red (I’) in an *Abl*^*2*^*/Abl*^*4*^ brain displaying an absence of α and β lobes. Note the small α and β branches in I. (J-J”) Expression of Abl within the MB using an *Abl-GFP* genomic transgene (J). α and β lobes are revealed by anti-Fas2 staining in red (J’). Merge of GFP and anti-Fas2 staining (J”). All panels correspond to 48 hAPF brains except for E and the rescue experiment in D which are from adult brains. White arrowheads show the peduncle or common part of the αβ axon, white arrows show the αβ branch point, yellow arrowheads show the α axon branch or the α lobe and pink arrowheads show the β axon branch or the β lobe. The scale bar in panels A-C and F-J indicates 30 μm. Images are composite stacks to allow the visualization of axon trajectories along their entire length. Full genotypes: (A) wild type: *y w*^*67c23*^. (B and C) *y w*^*67c23*^*;; Abl*^*2*^ *FRT2A / Abl*^*4*^ *FRT2A*. (D) top to bottom: *y w*^*67c23*^*;; Abl*^*2*^ *FRT2A / Abl*^*1*^ *FRT2A*. *y w*^*67c23*^*; Abl-GFP / +; Abl*^*2*^ *FRT2A / Abl*^*1*^ *FRT2A*. (E) top to bottom: *y w*^*67c23*^ */* Y*; UAS-Abl / UAS-mCD8-GFP;; OK107-GAL4 / +*. *y w*^*67c23*^ */* Y*; UAS-Abl*^*KD*^ */ UAS-mCD8-GFP; TM6B*,*Tb*^*1*^ */ +; OK107-GAL4 /* +. (F) w* tubP-GAL80 hs-FLP122 FRT19A / w* sn FRT19A; c739-GAL4 UAS-mCD8-GFP / UAS-mCD8-GFP. (G-H-I) w* tubP-GAL80 hs-FLP122 FRT19A / w* sn FRT19A; c739-GAL4 UAS-mCD8-GFP / UAS-mCD8-GFP; Abl^*2*^ */ Abl*^*4*^. (J) *y w*^*67c23*^*; Abl-GFP / +*.

The phenotype of MB αβ neurons with alterations in *Abl* expression has not previously been described thoroughly. We have used three *Abl* alleles (*Abl*^*1*^, *Abl*^*2*^ and *Abl*^*4*^) to characterize the requirement of *Abl* function in MB morphology (S2A Fig). Each of three double heterozygous combinations (*1/2*, *2/4* and *1/4*) are largely adult lethal but are viable at 48 hours after puparium formation (hAPF) enabling their effects to be investigated at that stage when the αβ neurons are present [37,40]. Interestingly, all the three allelic combinations gave similar MB phenotypes with a mixture of wild-type (WT) lobes (~20%) and MB lacking α-lobes, β-lobes, or both (~80%) (Fig 3A–3D). These *Abl* mutant phenotypes, including the adult lethality, were significantly rescued by introducing the *Abl-GFP* genomic transgene (from 20% WT MBs to 80%, P < 10^−5^; Fig 3D). *Abl* is known to be toxic when expressed in an unregulated fashion [21,25], and to cause axon growth and guidance defects when overexpressed in some neurons [29–31]. We therefore overexpressed *Abl* specifically in the MBs using the GAL4/UAS system [41]. Overexpression of WT *Abl* in MB neurons with the *OK107-GAL4* driver produced αβ lobe loss phenotypes similar to those observed with *Abl* LOF alleles (Figs 3E and S2B–S2E). Note that the use of a the weaker *c739-GAL4* driver resulted in no lobe loss phenotype (n = 100). The penetrance of the gain-of-function (GOF) mutant phenotype was even higher than that in the *Abl* LOF alleles; WT MBs were never detected. In contrast, expression of a kinase dead version of *Abl* failed to produce a MB mutant phenotype, although expression levels of WT and kinase-dead Abl from transgenes were similar (S2F–S2G Fig). Taken together, these data demonstrate that the expression of *Abl* and its kinase activity must be tightly controlled in the MBs in order to ensure normal MB αβ axon morphology. In order to determine if other MB neurons, in addition to the αβs, are sensitive to the overexpression of Abl we have also examined at the α’β’ and the γ MB adult neurons. The α’β’s were clearly affected displaying no WT MBs out of 93 MBs (S2 Fig) although the adult γ looked essentially wild-type (2 MBs with “round shape” γ out of 93 MBs). Therefore, both αβ and α’β’ are sensitive to Abl overexpression.

### *Abl* function is required for MB axon outgrowth and is expressed in MB αβ neurons

The absence of MB lobes can result from either growth or guidance defects [5,40]. In order to establish which of these two cellular phenomena is affected by loss of *Abl*, we first produced neuroblast mutant MARCM clones, which encompass hundreds of neurons, with the three different *Abl* alleles. These clones appeared to be predominantly wild-type with only between 4% to 11% showing the absent lobe phenotype (Table 1A). Our observation of highly-expressive lobe defects in the MBs of animals that are fully mutant for *Abl* versus the relative lack of phenotypes in MBs containing *Abl* mutant clones is consistent with the evidence cited above that Wnt-PCP acts non-cell autonomously for MB axon outgrowth [38], and that Abl acts downstream of Appl to promote this mechanism. However, given the low percentage of *Abl* mutant MARCM clones that displayed morphological defects we were not able to go further in studying single-cell mutant clones. We therefore produced fully mutant *Abl*^*2*^*/Abl*^*4*^ animals, and in them, we generated clones that express mCD8-GFP in single cells or small groups of cells. We term these “visualization clones” since only the membrane marker is clonal, and not the *Abl* mutation. For this experiment, clones are initiated in L3 larvae, and examined at 48 hAPF (to avoid the substantial late-pupal/adult lethality of homozygous *Abl* mutations). Two-cell/single cell visualization clones in *Abl* mutant animals revealed highly-penetrant growth defects (87% n = 15) (Table 1B and Fig 3F–3I’). We conclude that *Abl*, like *Appl*, is required for MB αβ axon outgrowth. To visualize the localization of Abl protein we employed a transgenic fly bearing a genomic *Abl-GFP* construct, which rescues both *Abl* mutant lethality and the mutant MB phenotypes (see above) and therefore is a bona fide endogenous marker for Abl. *Abl-GFP* is expressed broadly and homogenously in the brain from L3 to adult with elevated levels in the MB αβ axons of 48 hAPF brains (Fig 3J–3J”). Taken together, these data show that *Abl* is expressed in the MBs and that *Abl* function is required for MB αβ axon outgrowth. Additional neuroblast and more than two-cell visualization clones in *Abl* mutant animals confirmed the Abl requirement for MB αβ axon outgrowth (S3 Fig). In order to know if other MB neurons, in addition to the αβ’s, are sensitive to the lack of Abl we have also looked at the α’β’ and the γ MB neurons. We could not assess the α’β’ neurons because neither the anti-Trio antibody nor the *c305a-GAL4* line labelled the MB α’β’ neurons adequately before 48 hAPF. However, anti-Fas2 revealed a clear defect in *Abl*^*2*^*/Abl*^*4*^ γ neurons (S3 Fig). Therefore, at least the γ and the αβ MB neurons are sensitive to the lack of Abl function. Since both αβ and α’β’ are sensitive to Abl overexpression (see above), all MB neurons appear to require normal levels of Abl function.

**Table 1:** *Abl* mutant MARCM clones.

(A) *Abl*^*mut*^/ *Abl*^*mut*^ MARCM clones.					
Neuroblast clones					
Genotype	WT	Absence of α lobe	Absence of β lobe	Absence of α/β lobes	n
Control	**22** 100%	**--**	**--**	**--**	22
*Abl*^*2*^ / *Abl*^*2*^	**43** 90%	**5** 10%	**--**	**--**	48
*Abl*^*4*^ / *Abl*^*4*^	**37** 82%	**5** 11%	**3** 7%	**--**	45
*Abl*^*1*^ / *Abl*^*1*^	**71** 92%	**3** 4%	**3** 4%	**--**	77
(B) Visualization *Abl*^*2*^*/Abl*^*4*^ MARCM clones.					
Two-cells / Single-cell clones					
Genotype	WT	α branch growth defect	β branch growth defect	α/β branch growth defects	n
Control	**30** 100%	**--**	**--**	**--**	30
*Abl*^*2*^ */ Abl*^*4*^	**2** 13%	**10** 67%	**--**	**3** 20%	15
>Two-cells clones					
Genotype	WT	Absence of α lobe	Absence of β lobe	Absence of α/β lobes	n
Control	**9** 100%	**--**	**--**	**--**	9
*Abl*^*2*^ */ Abl*^*4*^	**6** 23%	**14** 54%	**2** 8%	**4** 15%	26
Neuroblast clones					
Genotype	WT	Absence ofα lobe	Absence of β lobe	Absence of α/β lobes	n
Control	**9** 100%	**--**	**--**	**--**	9
*Abl*^*2*^ */ Abl*^*4*^	**2** 17%	**7** 58%	**--**	**3** 25%	12

WT: wild-type clones. n: number of clones analyzed.

Full genotypes: A) Control: *y w*^*67c23*^ *hs-FLP122* / +; *c739-GAL4 UAS-mCD8-GFP* / +; *tubP-GAL80*, *FRT2A* / *FRT2A*. Mutant:. *y w*^*67c23*^ *hs-FLP122* / +; *c739-GAL4 UAS-mCD8-GFP* / +; *tubP-GAL80*, *FRT2A* / *Abl*^*2*^ *FRT2A*. *y w*^*67c23*^ *hs-FLP122* / +; *c739-GAL4 UAS-mCD8-GFP* / +; *tubP-GAL80*, *FRT2A* / *Abl*^*4*^ *FRT2A*. *y w*^*67c23*^ *hs-FLP122* / +; *c739-GAL4 UAS-mCD8-GFP* / +; *tubP-GAL80*, *FRT2A* / *Abl*^*1*^ *FRT2A*. B) Control: w* tubP-GAL80 hs-FLP122 FRT19A / w* sn FRT19A; c739-GAL4 UAS-mCD8-GFP / UAS-mCD8-GFP. *Abl*^*2*^*/Abl*^*4*^: w* tubP-GAL80 hs-FLP122 FRT19A / w* sn FRT19A; c739-GAL4 UAS-mCD8-GFP / UAS-mCD8-GFP; Abl^*2*^ */ Abl*^*4*^.

### *htt* modifies the *Abl* mutant phenotype in MB axon outgrowth

**Fig 4:**
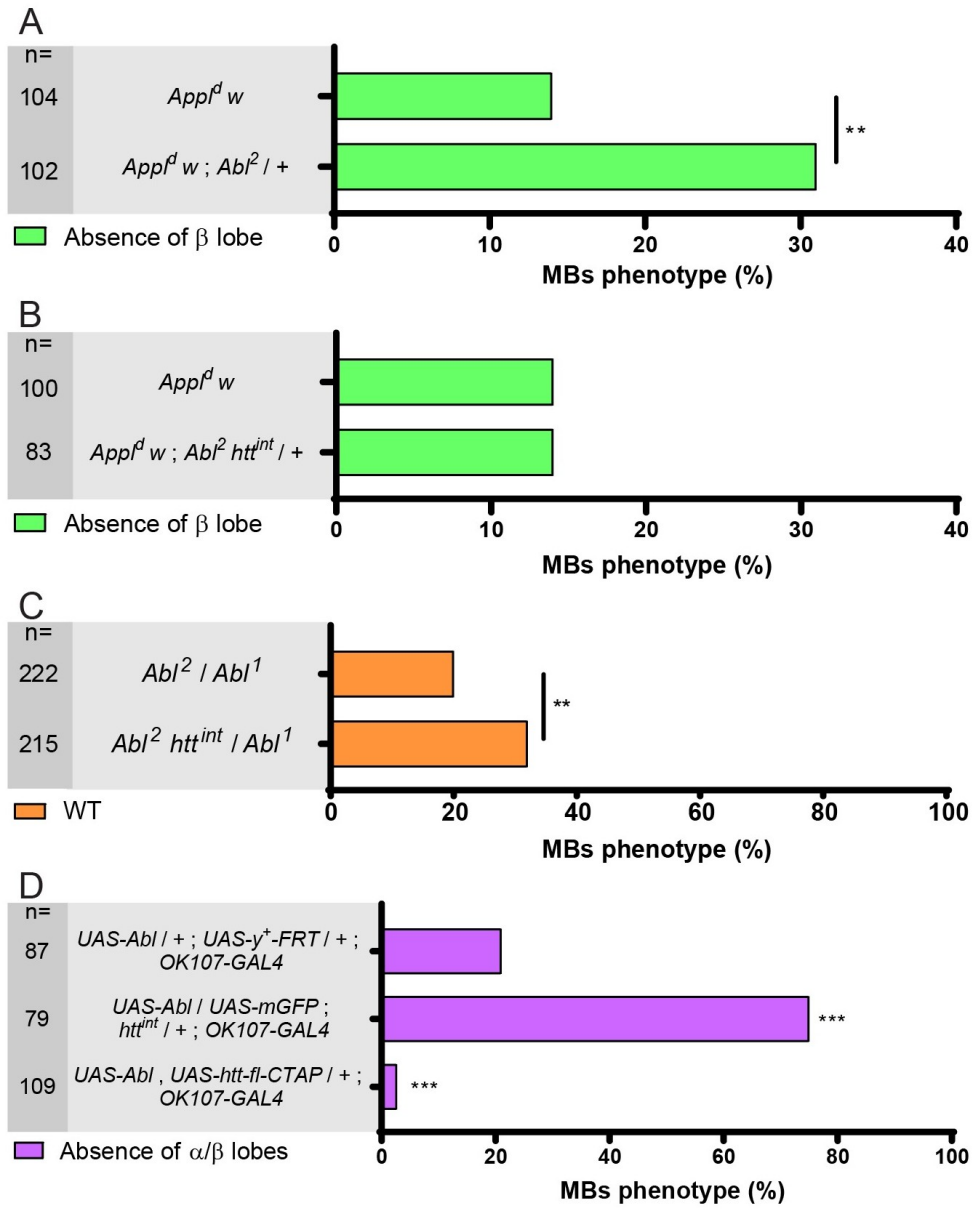
*htt* interacts with *Abl*. (A) The *Appl*^*d*^ mutant phenotype is enhanced in an *Abl*^*2*^/+ mutant background. (B) However, the *Appl*^*d*^ mutant phenotype is not modified in an *Abl*^*2*^ *htt*^*int*^/*+* + mutant background. (C) The *Abl*^*2*^/*Abl*^*1*^ mutant phenotype is partially rescued by the loss of one copy of *htt* as inferred by the increase of the wild-type (WT) MBs. (D) The absence of α and β lobe phenotype observed in *UAS-Abl* driven by *OK107-GAL4* is strongly increased by the loss of one copy of *htt* (upper and middle bars). However, this phenotype is rescued by the overexpression of full-length *htt* (upper and lower bars). n = number of MBs analyzed, ** P<0.01 and *** P < 0.001. Significance was calculated by a Chi^2^ test for A-C and by a multiple comparison Fisher’s exact test (P = 3.4 10^−28^) followed by post-hoc Bonferroni’s multiple comparison correction (p-values = 6.3 10^−12^ and 0.0003 for D). All panels correspond to adult brains except for B which is from 48 hAPF brains. Full genotypes: (A) top to bottom: *Appl*^*d*^ w* / Y*; c739-GAL4 UAS-mito-GFP / +*. *Appl*^*d*^ w* / Y*; c739-GAL4 UAS-mito-GFP /* +; *Abl*^*2*^ */ +*. *Appl*^*d*^ w* / Y; *c739-GAL4 UAS-mito-GFP / +*; *Abl*^*2*^ *FRT2A htt*^*int*^ */ +*. (B) top to bottom: *y w*^*67c23*^*;; Abl*^*2*^ *FRT2A / Abl*^*1*^ *FRT2A*. *y w*^*67c23*^*;; Abl*^*2*^ *FRT2A htt*^*int*^ */ Abl*^*1*^ *FRT2A*. (C) top to bottom: *y w*^*67c23*^ / Y; *UAS-Abl / +; UAS-FRT-y+-FRT / +; OK107-GAL4 / +*. *y w*^*67c23*^ / Y; *UAS-Abl / UAS-mCD8-GFP; htt*^*int*^ */ +; OK107-GAL4 / +*. *y w*^*67c23*^ / Y; *UAS-Abl*, *UAS-htt-fl-CTAP / +;; OK107-GAL4 /* +. Note that *UAS-FRT-y+-FRT* and *UAS-mito-GFP* are used here as neutral *UAS* to adjust for 2 *UAS* sequences in the three different genotypes.

As discussed above, the *Appl*^*d*^ MB phenotypes in β axons can be rescued by UAS-driven overexpression of Abl [5], and both the *Appl*^*d*^ and *dsh*^*1*^ β axonal phenotypes can be rescued by modest overexpression of *Abl* using a genomic transgene, (S1B and S1C Fig). Taken together with the finding that *htt* suppresses both *Appl* and *dsh* MB phenotypes, we hypothesized that *htt* might be a suppressor of *Abl* action. To test this hypothesis, we conducted three sets of experiments to examine the effects of reducing *htt* dosage on *Abl* phenotypes. First, we found that the increased severity of the MB mutant phenotype in *Appl*^*d*^; *Abl*^*2*^/+ individuals was completely abolished when one copy of *htt* was also removed (31% absence of β lobe in *Appl*^*d*^; *Abl*^*2*^/+ vs 14% in *Appl*^*d*^; *Abl*^*2*^ *htt*^*int*^/+ compared to 14% in *Appl*^*d*^; +/+—Fig 4A upper and lower bars). Second, a modest but significant increase in the proportion of WT MBs in *Abl*^*2*^*/Abl*^*1*^ individuals was observed when one dose of *htt* was removed (from 20% to 32%—Figs 4C and S4). Third, enhancement of the *Abl* GOF mutant phenotype, measured by the simultaneous absence of both α and β lobes, was seen when one copy of *htt* was removed (from 21% to 75%—Fig 4D upper and middle bars). Conversely, rescue of this phenotype was observed when *htt* was overexpressed using *UAS-htt-fl-CTAP* driven by *c739-GAL4* (from 21% to 3%—Fig 4D upper and lower bars). This interpretation of a phenotypic rescue is further supported in this comparison by the increase in the number of wild-type MBs (0% in *UAS-Abl*, vs 16% in *UAS-Abl*; *UAS—htt-fl-CTAP*). The ability of *htt/*+ to suppress the *Abl* LOF phenotype or to enhance the *Abl* GOF phenotype, as well as the ability of *htt* overexpression to suppress the *Abl* GOF phenotype appears to correlate with the amount of kinase-competent residual protein in the various mutant backgrounds (see Discussion). Taken together, these data strongly indicate that Htt is a repressor of Abl function during MB axon outgrowth.

**Fig 5:**
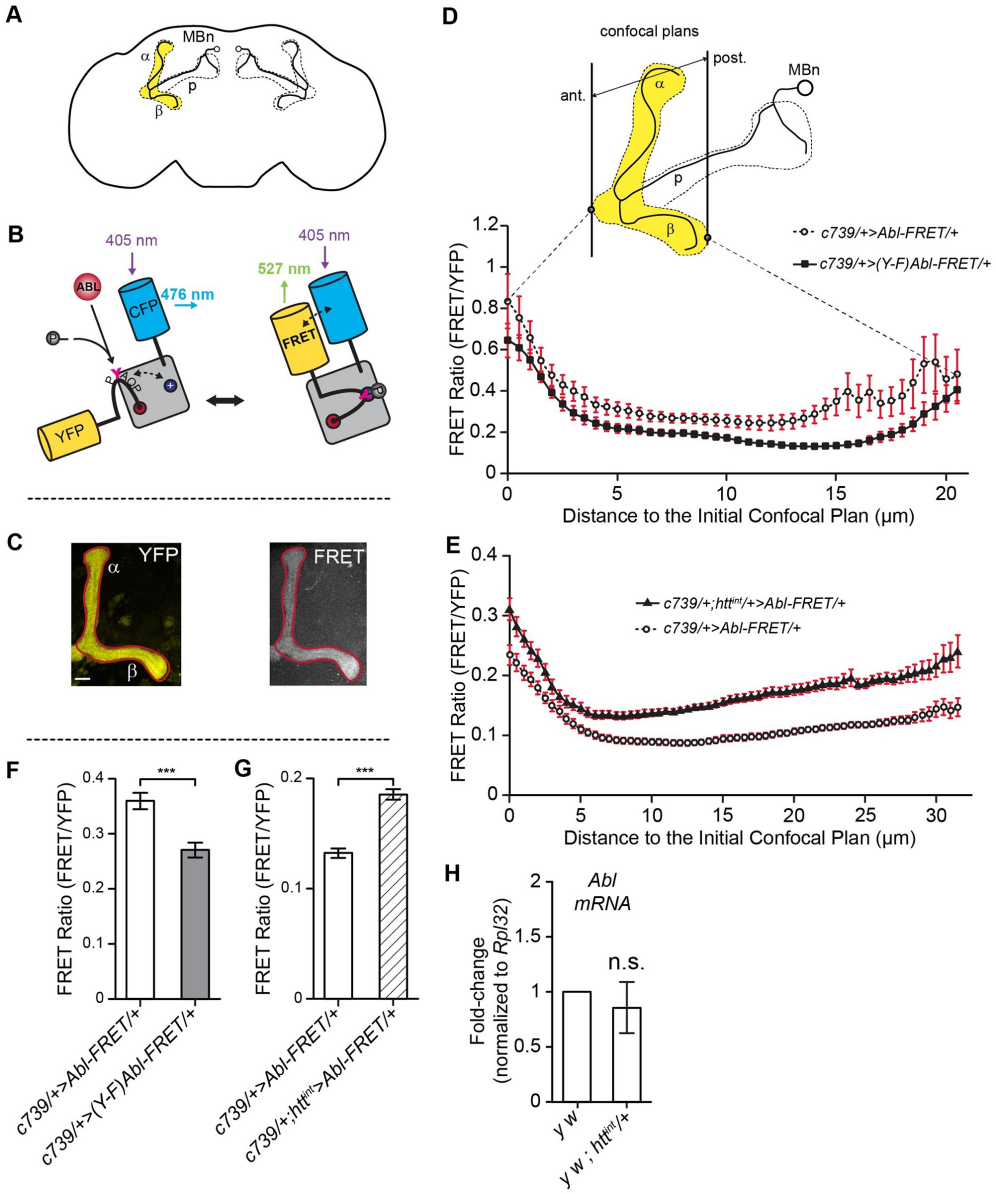
Reducing *htt* expression increases *Abl*-FRET biosensor phosphorylation state during MB development. (A) Schematic representation of an imaged MB in the brain. (B) The *Abl*-FRET biosensor is based on mammalian CRK protein scaffold with two additional fluorescent proteins (CFP and YFP). ABL kinase activity induces phosphorylation of *UAS-Abl*-FRET biosensor leading to its spatial rearrangement and increased FRET efficiency [59]. (C) Representative maximal projection of the YFP and FRET signals recorded in the MB lobes of adult flies using confocal microscopy. These are presented as examples of the kind of image data that goes into the FRET ratio calculation. Scale bar: 10μm. (D) FRET and YFP signals are recorded on adjacent 0.5 μm confocal planes along all the anterior-posterior axis of the α and β MB lobes in adult flies. Either wild-type *UAS-Abl*-FRET biosensor or a mutated form (*UAS-Y➔F Abl-*FRET) are expressed in the αβ MB neurons using the *c739-GAL4* driver. In the *UAS-Y➔F Abl-*FRET biosensor, the tyrosine located in ABL target site (PYAQP) was replaced by a phenylalanine (PFAQP) impairing phosphorylation [42]. FRET efficiency is significantly reduced for *UAS-Y➔F Abl-*FRET relative to *UAS-Abl*-FRET biosensor for all confocal planes considered except for the nine most anterior and six most posterior planes. Two-tailed Mann-Whitney tests on non-Normally distributed data. Results are mean ± SEM with n ≥ 7 MB for each confocal plane. (E) Reducing *htt* expression increases FRET efficiency of *UAS-Abl*-FRET biosensor in third instar larval MB lobes. The *UAS-Abl*-FRET biosensor is expressed in MB neurons using the *c739-GAL4* driver in control (+/+) and *htt*^*int*^/+ flies. FRET efficiency is significantly increased in *htt*^*int*^/+ flies for all confocal planes along the anterior-posterior axis except for the three located 3 to 4 μm from the initial confocal plane. Two-tailed Mann-Whitney tests on non-Normally distributed data. Results are mean ± SEM with n ≥ 13 MB for each confocal plane. (F) FRET efficiency is globally reduced in *UAS-Y➔F Abl-*FRET mutant versus *UAS-Abl*-FRET. FRET efficiency is averaged for all confocal planes and all along the anterior-posterior axis. Two-tailed Mann-Whitney test with non-Normally distributed data. Results are mean ± SEM with n ≥462; *** p<0.001. (G) FRET efficiency is globally increased when *htt* expression is reduced. FRET efficiency is averaged for all confocal planes and all along the anterior-posterior axis. Two-tailed Mann-Whitney test on non-Normally distributed data. Results are mean ± SEM with n ≥ 1055; *** p<0.001. (H) *Abl* mRNA expression is not changed in L3 brains following *htt* partial inactivation. *Abl* expression was assessed using RT-qPCR in L3 brains of *htt*^*int*^ /+ versus WT (+/+) male flies. Results show three independent biological replicates. Full genotypes: Genotypes: *y w*^*67c23*^ / Y; *c739-GAL4*/+; *UAS-Abl-FRET/+*. *y w*^*67c23*^ / Y; *c739-GAL4*/+; *UAS-Y-F Abl-FRET/+*. *y w*^*67c23*^ / Y; *c739-GAL4*/+; *htt*^*int*^ *UAS-Abl-FRET/+*.

### Reduction of *htt* increases Abl kinase activity in the developing MBs

**Fig 6:**
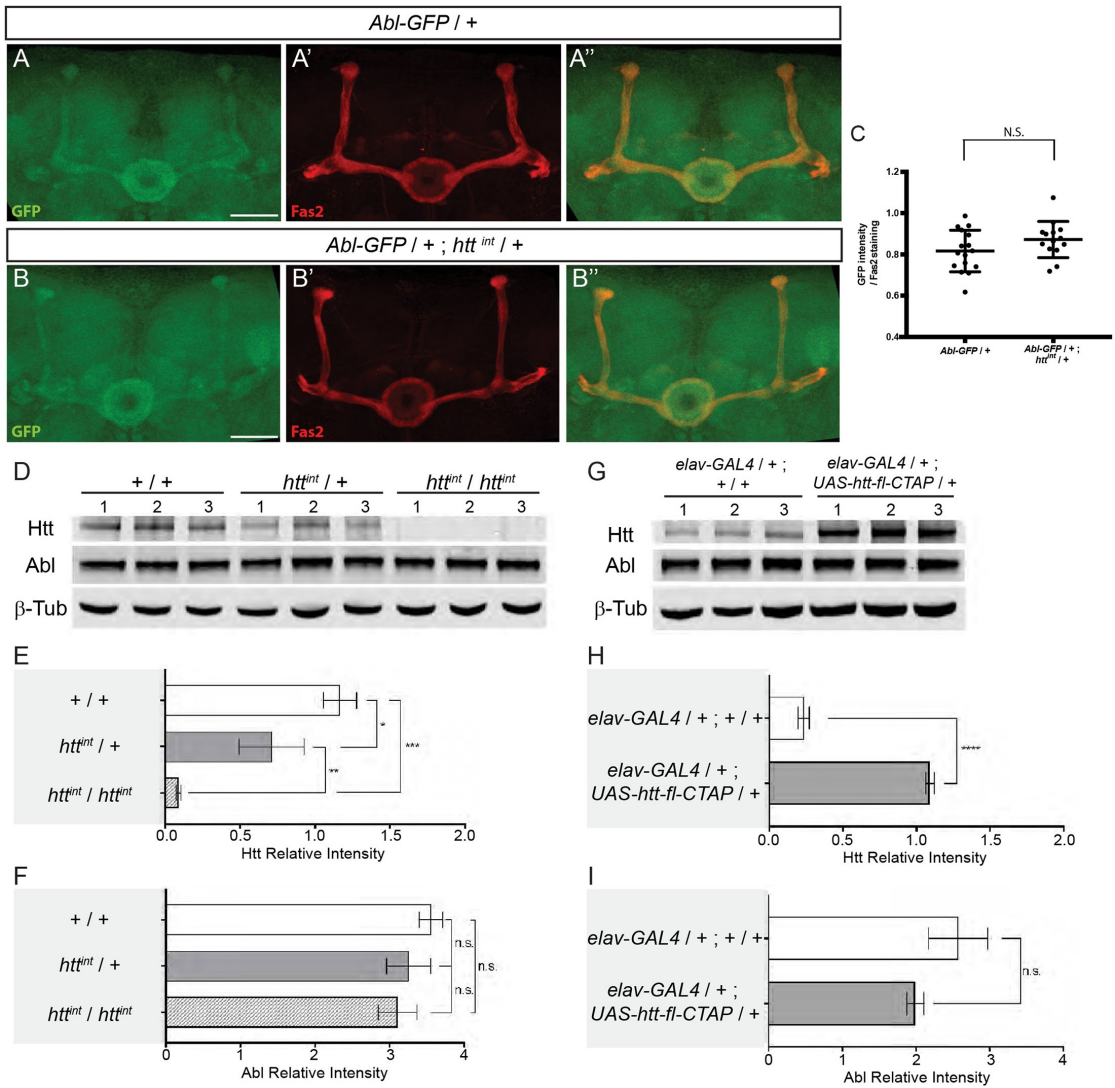
*htt* does not seem to affect the quantity of ABL in the MBs or the total levels of neuronal Abl in *Drosophila*. (A-B”) Expression of the *Abl-GFP* genomic transgene in a WT (A) and in a *htt*^*int*^ heterozygous mutant background (B) at 48 hAPF. Anti-Fas2 staining marked αβ neurons (A’-B’). Merge of GFP and anti-Fas2 staining (A”-B”). Note that panels A-A” are also presented in Fig 3J–3J”. (C) Quantitation of the GFP expression within MBs is not significantly different (N.S.) between WT and *htt*^*int*^ heterozygous mutant background using a Mann-Whitney *U* test. The scale bar indicates 30 μm. Details of image quantification procedure and full genotypes are: (C) After having outlined the MB with the Fas2 staining, GFP and Fas2 intensities from MB shape were quantified for each slices of the stack. The GFP intensity of each slices was averaged and then normalized by the mean Fas2 intensity. Number of MB analyzed: control = 16, *htt* mutant = 14. Quantitation of the GFP expression within MBs is not significantly different between WT and *htt*^*int*^ heterozygous mutant background using a Mann-Whitney *U* test. Quantitation were done with ImaJ software. Images are composite stacks to allow the visualization of axon trajectories along their entire length. Genotypes: (A) *y w*^*67c23*^*; Abl-GFP / +*. (B) *y w*^*67c23*^*; Abl-GFP / +; htt*^*int*^ */ +*. (D) Western blots of whole cell lysates from adult heads from the following genotypes: +*/*+, *htt*^int^/+ and *htt*^*int*^*/htt*^*int*^. Levels of Htt, Abl, and β-Tubulin (β-Tub) were assessed by probing blots using the indicated antibodies. 1, 2, and 3 indicate biological replicates for each line. (E and F) Quantitation of Htt (E) or Abl (F) protein levels in the indicated genotypes was assessed from western blots in (D) relative to β-Tubulin and plotted as relative band intensity. (G) Htt was over-expressed using *elav* ^*c155*^*-GAL4 > UAS-htt-fl-CTAP*. Lysates from adult heads were subjected to western blotting to assess Htt, Abl, and β-Tubulin levels. 1, 2, and 3 indicate biological replicates for each line. (H and I) Quantitation of Htt (H) or Abl (I) protein levels in the indicated genotypes was assessed from western blots in (G) relative to β-Tubulin and plotted as relative band intensity. In E and F, significance was calculated by one-way ANOVA. In H and I, significance was calculated by unpaired *t*-test. In E, F, H and I, errors indicate standard deviation. (n.s. not statistically different, *P<0.05, ** P<0.01, *** P<0.001,**** P<0.0001).

One potential explanation for the suppressor effect of *htt* on the *Abl* mutant MB phenotype is that Htt inhibits Abl kinase activity. To test this idea, we employed a fluorescence resonance energy transfer (FRET) biosensor probe that allows Abl kinase activity to be assayed *in vivo* in *Drosophila* [42]. Using this tool, we first showed that Abl kinase activity is detectable in the MBs by comparing the FRET activity of wild type *UAS-Abl*-FRET versus the FRET activity of a mutant *UAS-Y➔F Abl-*FRET that lacks its phosphorylatable tyrosine (Figs 5D–5F and S5). We then tested the FRET efficiency in the developing MBs of control (+/+) versus *htt*^*int*^/+ larvae and detected a significant increase of the FRET efficiency when one copy of *htt* was removed (Fig 5E and 5G). Note that the exact value of the FRET ratio depends on various microscope and laser settings during imaging. Consequently, this number can only be compared directly between samples imaged together in a single imaging session and cannot be compared from separate experiments. In these experiments, images for validation of the FRET(WT) probe by comparison to FRET(Y->F) were collected in a single session (D,F) as were images comparing FRET activity in a wild type vs *htt/+* genetic background (E,G). Quantitative PCR (qRT-PCR) analysis of control versus *htt*^*int*^*/*+ larval brains revealed no difference in *Abl* mRNA expression (Fig 5H). Furthermore, there were no apparent differences in the overall levels of Abl protein in the *htt*/+ heterozygous MBs relative to WT controls (Fig 6A–6C). The expression level of Abl protein is fairly homogenous in the larval brain rendering the quantitation of Abl in the MBs difficult. In order to reliably quantitate Abl protein levels, we measured the level of Abl protein in 48 hAPF MBs. As noted above, Abl is enriched during this stage. Finally, reduction or increase in Htt expression did not alter the total levels of neuronal Abl protein in *Drosophila* heads (Fig 6D–6I). Taken together, these data strongly suggest that Htt is a repressor of Abl kinase activity in the developing MB axons.

## Discussion

Tumorigenesis and neurodegeneration may be two sides of the same coin [43]. Indeed, defining the overlap of molecular pathways implicated in cancer and neurodegeneration may open the door to novel therapeutic approaches for both groups of disorders [43]. Correlative studies have highlighted a decreased cancer incidence in the population with the neurodegenerative disorder Huntington’s disease and both wild-type and mutant huntingtin (Htt) have been implicated in tumor progression [44]. Interestingly, it has been proposed that, in the normal physiological situation, the neurodegeneration-related Amyloid precursor protein (APP) recruits the oncogenic Abelson (Abl) kinase in order to promote axonal outgrowth [5]. It is, therefore, tempting to propose that different neurodegenerative diseases (ND) may share components and mechanisms and that Abl may also have a role in ND.

In this study, we have shown that *Abl* is required for axonal growth in the MBs, a brain structure that is involved in memory. Furthermore, we show that both *Abl* overexpression and lack of expression in the MBs result in similar phenotypes, indicating the need to tightly regulate Abl activity during MB axon outgrowth. This raises the question of how Abl activity is normally negatively regulated during MB axon outgrowth. We confirmed the previous observation that overexpression of *Abl* rescues the *Appl*^*d*^ MB phenotypes [5]. Furthermore, we found that *Abl* overexpression also rescues the *dsh*^*1*^ MB phenotype. These two results support the model that Appl activates Abl, which in turn phosphorylates Dsh. At the genetic level, we expected an increase of Abl activity in an individual bearing a loss-of-function mutation of a putative *Abl* repressor. We therefore hypothesized that reducing the levels of an *Abl* repressor would result in suppression of the *Appl* and *dsh* mutant MB phenotype. We found that Htt is such an inhibitor of Abl activity in the MBs. The loss of one dose of *htt* increased the activity of the wild-type Abl still present in *Abl*^*2*^*/*+ individuals and therefore prevented the enhancement of the MB mutant phenotype. While a number of studies have concluded that Htt deficiency results in significant alterations to kinase signaling pathways [45], to our knowledge this study is the first to implicate the crucial tyrosine kinase, Abl. Together, these data demonstrate the power that neurodevelopmental studies have to reveal close functional relationships between genes implicated in different forms of neurodegenerative disease.

It was a surprise that null *htt* mutants show no obvious developmental defects in *Drosophila* although strong defects could have been expected from the lack of such a conserved protein [12]. One possible hypothesis is that, due to its fundamental importance, some functional redundancy has been selected to buffer against variation in the production of the Htt protein. We reasoned that altering the levels of another protein, particularly another protein known to be implicated in neurodegeneration, could reveal cryptic phenotypes of *htt* mutation during brain development. Following this reasoning, we combined a null *Appl* mutant with heterozygous null *htt* mutations in double mutant individuals. Unexpectedly, we found that mutant *htt* suppressed the *Appl* MB axonal outgrowth defect.

*Abl* is a key component of the *Appl* signaling pathway required for axonal arborization and growth in the fly brain and the functional relationship between these two proteins is likely conserved in mammals [5,34]. While the role of APP-mediated signaling has been shown most clearly in *Drosophila*, a number of lines of evidence suggest that mammalian APP also fulfills a signaling role [46]. Importantly, and in line with its proto-oncogenic role, *Abl* tyrosine kinase activity is tightly regulated by intramolecular inhibition [22]. Although *Abl* is clearly required as a downstream effector of *Appl* in the MB axon growth, its precise role and regulation in the MBs has not been described previously.

The three *Abl* alleles used in this study have all been shown to result in truncated proteins [47]. While *Abl*^*1*^ retains the SH3, SH2 and TK domains of Abl, *Abl*^*2*^ is mutated within the TK domain and only retains the SH3 and SH2 domains, while *Abl*^*4*^ is mutated in the SH2 domain, and only retains an intact SH3 domain. It therefore seems likely that very little or no residual *Abl* function remains in *Abl*^*2*^*/Abl*^*4*^ and *Abl*^*4*^*/Abl*^*1*^ individuals and may explain why no rescue was observed when one dose of *htt* was removed in these genetic backgrounds. In contrast, the *Abl*^*1*^*/Abl*^*2*^ allelic combination is likely less severe than the other two genotypes and *Abl*^*1*^*/Abl*^*2*^ animals do accumulate truncated Abl proteins. Indeed, while significant amounts of truncated *Abl*^*1*^ and *Abl*^*2*^ mutant proteins are detectable, only faint protein bands are observed in *Abl*^*4*^ pupae [48]. Therefore, some functionally significant kinase activity could remain in *Abl*^*2*^*/Abl*^*1*^ individuals and the loss of one dose of *htt* might increase the activity of the remaining kinase activity. Finally, removing one dose of *htt* in animals overexpressing Abl would result in even more Abl activity, which in turn would exacerbate the mutant phenotype. Contrarily, over-expressing Htt, as in the *UAS-Abl*, *UAS-htt* doubly overexpressing individuals, would inhibit Abl function when compared to the *UAS-Abl* overexpression alone which thus might explain the observed rescue.

**Fig 7:**
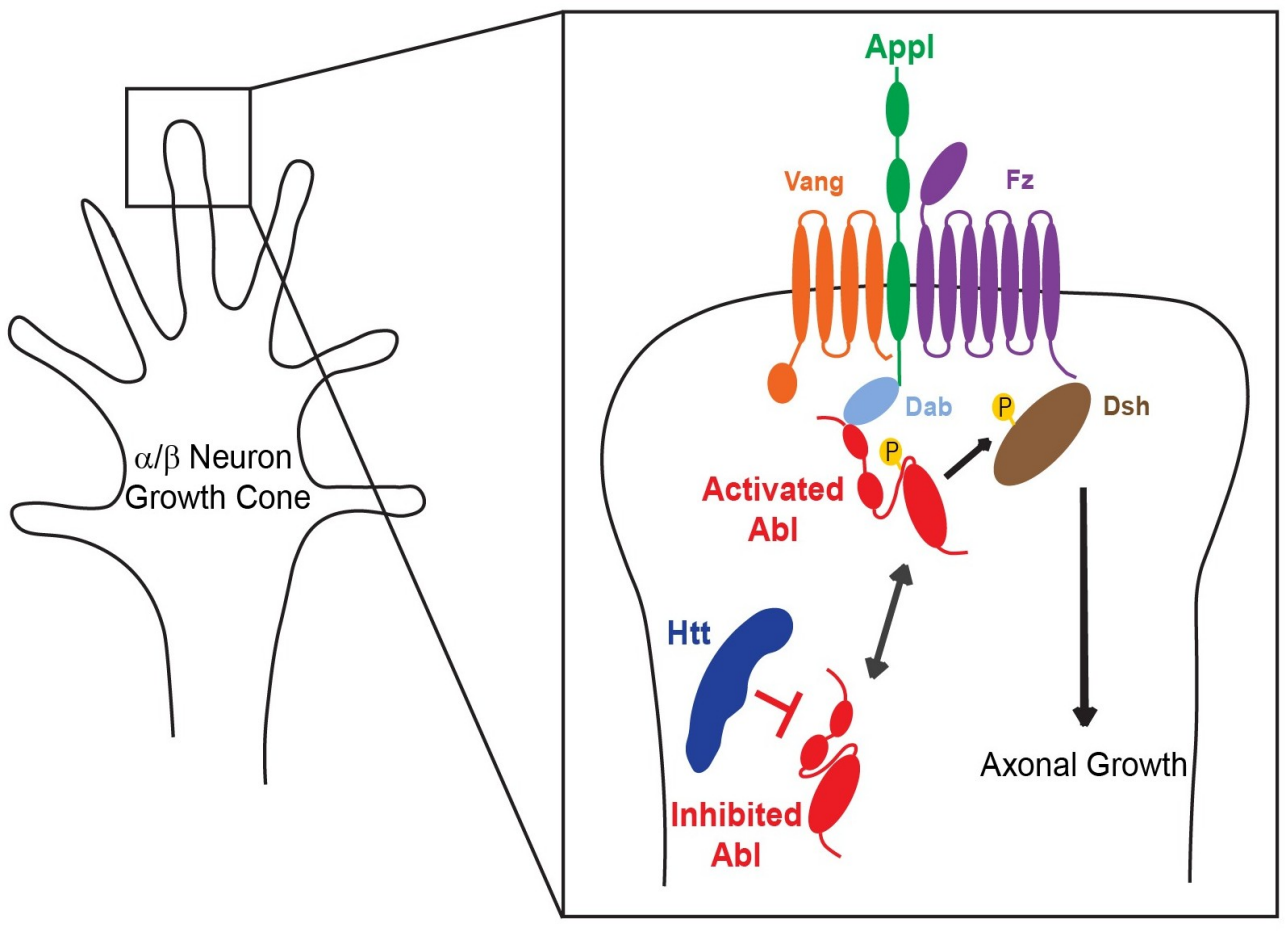
Htt is a repressor of Abl activity required for Appl-induced axonal growth. Schematic representation of the proposed mechanism. Htt represses Abl activity at the growth cone of αβ neuron in MB. Appl and Htt have opposing roles in MB development, to promote and suppress Abl kinase activity, respectively, to maintain the appropriate intermediate level of Abl phosphorylation necessary for axon growth. This model is based on the proposed model of Appl signaling required for MB axon growth (Fig 7 in [5]).

There are three different levels at which Htt might act to modulate *Abl* activity. First, Htt could either directly or indirectly affect *Abl* mRNA levels. Although fly Htt has been described as a cytoplasmic protein [12], *htt* has been shown to be a suppressor of position-effect variegation, suggesting a possible role in chromatin organization [13]. This hypothesis is unlikely for the MB phenotype described here since qRT-PCR analysis of third instar brains did not reveal a significant differences in *Abl* mRNA levels between *htt*^*int*^/+ and control individuals. Second, Htt could play a role regulating Abl protein level in the MBs. We also consider this unlikely since the quantity of the endogenous Abl is unchanged in *htt*^*int*^/+ relative to control individuals. Third, Htt could influence the kinase activity of Abl itself. Taking advantage of a FRET biosensor enabling Abl kinase activity to be assayed directly in the MBs, we revealed a significant increase in active Abl in *htt*^*int*^/+ versus control individuals. Therefore, we favor a model of Htt acting as an inhibitor of Abl kinase activity during normal MB axonal growth (Fig 7). Abl activity in axons needs to be maintained within rather narrow limits [32,33]. These two recent studies show that either increase or decrease of Abl activity cause disorganization of actin structure in the growth cone and prevent the orderly oscillation of growth cone actin that is the motor for growth cone advance and thus axon extension. Those papers also explain why *Abl* gain and loss can result in superficially similar mutant axon patterning phenotypes even though the molecular effects of Abl increase versus decrease are opposite.

The HEAT repeat domains of Htt are thought to function as a solenoid-like structure that acts as a scaffold and mediates inter- and intra-molecular interactions [9]. It is therefore tempting to propose that a scaffolding role of Htt could elicit repression of Abl kinase activity. At least in the MBs, a balance seems to exist in the activity of Abl, positively regulated by the membrane complex formed by the core PCP proteins and Appl, and suppressed by Htt and possibly other proteins, as well. On one hand, if Appl is absent, Abl is not optimally activated leading to defects in MB axon growth. Conversely, decrease of Htt to 50% of wild type levels leads to de-repression of *Abl* kinase activity, which in turn compensates for its sub-optimal level of activation in the absence of *Appl*. This unexpected apparent balance of activation and inhibition of *Abl* by *Appl* and *htt*, whose mutant orthologs are central players in human neurological disease, may define a conserved functional interaction to maintain Abl activity in the relatively narrow window to appropriately effect axon outgrowth.

## Material and methods

### *Drosophila* stocks

All crosses were performed on standard culture medium at 25°C. Except where otherwise stated, all alleles and transgenes have been described previously (http://flystocks.bio.indiana.edu/). The following alleles were used: *Abl*^*1*^, *Abl*^*2*^, *Abl*^*4*^, *Appl*^*d*^, *dsh*^*1*^, *htt*^*int*^ [13], *htt-ko* and *Df-htt* [12]. The following transgenes were used: *UAS-Abl* (Bloomington Stock Center line (BL) #28993), *UAS-Abl*.*K417N* (from BL #8566) named here *UAS-Abl*^*KD*^ for kinase dead, *UAS-Abl*-FRET and *UAS-Y➔F Abl-*FRET [42], *UAS-RNAi-htt* [49], *UAS-mCD8GFP*, *UAS-mito-GFP*, *UAS-FRT-y*^*+*^*-FRT* and the genomic transgenic *Abl-GFP* [50]. *UAS-htt-fl-CTAP* was produced for this study (see Constructs). We used three GAL4 lines: *c739-GAL4* and *OK107-GAL4* expressed in MB neurons [51] and the pan-neuronal driver *elav*^*c155*^*-GAL4* (BL #458). Recombinant chromosomes were obtained by standard genetic procedures and were molecularly verified when required.

### Adult and pupal brain dissection and immunostaining

Adult brains were dissected in PBS after fly heads and thoraxes had been fixed for 1hr in 3.7% formaldehyde in PBS. They were then treated for immunostaining as previously described [52,53]. Pupal brains were dissected in PBS and fixed for 20min in 3.7% formaldehyde in PBS at 4°C with gentle rocking. After washing twice in PBS with 0.5% Triton X-100 (PBT) for 15 min at room temperature, they were incubated in PBT and 5% bovine serum albumin (BSA) (blocking solution) at room temperature for 30 min, followed by overnight incubation at 4°C with primary antibody diluted in blocking solution. Brains were then washed three times in PBS for 20 min, followed by 30 min in the blocking solution, and then addition of the secondary antibody with incubation for 3 hr at 4°C. Brains were then washed three times in PBS for 20 min and were mounted with Vectashield (Vector Laboratories). Antibody combinations used: anti-Fas2 (mAb 1D4 from DSHB) at 1:50 dilution followed by anti-mouse Cy3 (Jackson ImmunoResearch) at 1:300; rabbit anti-Myc (Cell Signaling) at 1:1000 followed by anti-rabbit Cy5 (Jackson ImmunoResearch) at 1:300; mouse anti-TAP (Santa Cruz Biotechnology) at 1:300 followed by anti-mouse Cy3 (Jackson ImmunoResearch) at 1:300; rabbit anti-Trio (kind gift from Barry Dickson) at 1:1000 followed by anti-rabbit Cy2 (Jackson ImmunoResearch) at 1:300. Anti-Fas2 (mAb 1D4 from DSHB) at 1:10 dilution followed by anti-mouse Alexa 647 (Jackson ImmunoResearch) at 1:300.

### Quantitation of the absence of mushroom body lobes

We took particular care in order to ascertain a suppression effect from a penetrance of about 15%. The absence of lobes was assessed with the anti-Fas2 staining or with the *c739-GAL4 UAS-mito-GFP* marker visualized with an epi-fluorescence microscope (Leica DM 6000). In order to be certain that we were indeed measuring suppression of the *Appl*^*d*^ or *dsh*^*1*^ 15–20% of absence of β lobes and not a mere variation from this rather low phenotypic penetrance (Figs 1, 2, 4, S1B and S1C), we followed a strict protocol. A large number (at least 50) of *Appl*^*d*^ *w* or *w dsh*^*1*^ or *Appl*^*d*^ *w dsh*^*1*^ females were collected, pooled together and crossed in groups of 25 with either *y w*^*67c23*^ wild-type control or *y w*^*67c23*^; *mutant*/*Balancer* males. In this way, there were always *Appl*^*d*^ *w*/Y or *w dsh*^*1*^/Y or *Appl*^*d*^ *w dsh*^*1*^/Y males from the same experiment that show the expected mutant phenotype, and any modifier in the *Appl*^*d*^ or *dsh*^*1*^ stocks, if it exists, should be statistically equally present in the control and in the experiment. We could therefore associate the suppression phenotype seen in *Appl*^*d*^ *w*/Y or *w dsh*^*1*^/Y or *Appl*^*d*^ *w dsh*^*1*^/Y; *htt*^*mutant*^ /+ males unequivocally to the presence of the *htt*^*mutant*^ allele. Noticeably, throughout this study in a total of 26 experiments, MBs from *Appl*^*d*^; *c739-GAL4 UAS-mito-GFP*/+ males were assessed. Of 3110 MBs, 451 (14.5%) displayed an absence of β lobe phenotype (22/141; 15/106; 16/84; 15/124; 15/105; 18/152; 19/108; 14/100; 14/100; 27/156; 25/164; 12/112; 2/36; 17/102; 21/139; 14/109; 19/158; 19/155; 15/104; 30/146; 14/91; 24/138; 13/124; 13/102; 11/90; 27/164). In addition, throughout this study, we assessed the absence of β lobe phenotype in wild-type controls. In a total of nine different experiments (0/106; 0/102; 0/100; 1/107; 0/100; 0/50; 0/50; 0/37; 1/107), control MBs displayed two MBs with absence of β lobes out of 759 MBs (0.26% showing absence of β lobes and 99.74% of the MBs appearing WT). Thus, wild-type flies almost invariably have intact MB lobes as was previously described [5].

### MARCM clonal analysis

The MARCM technique was used to generate clones in the MB [53]. We use the term MARCM clones when homozygous mutant clones were examined in a heterozygous background and visualization MARCM clones when homozygous mutant clones were examined in a homozygous mutant background. For MARCM neuroblast clones, L1 larvae were heat-shocked at 37°C for 1 hr and adult brains were dissected and stained. For visualization MARCM neuroblast clones, L1 larvae were heat-shocked at 37°C for 1 hr and 48 hAPF brains were dissected and stained. For visualization single and two-cell clones, L3 larvae were heat-shocked at 37°C for 15 min and 48 hAPF brains were dissected and stained.

### Microscopy and image processing

Images were acquired at room temperature using a Zeiss LSM 780 laser scanning confocal microscope (MRI Platform, Institute of Human Genetics, Montpellier, France) equipped with a 40x PLAN apochromatic 1,3 oil-immersion differential interference contrast objective lens. The immersion oil used was Immersol 518F. The acquisition software used was Zen 2011. Contrast and relative intensities of the green (GFP), red (Cy3) and blue (Cy5) channels were processed with Fiji Software. Quantitation was performed using ImageJ software.

### Constructs

*pUAS-htt-fl-CTAP*: A *htt* “mini-gene” bearing a dual C-terminal Tandem Affinity Purification tag (Protein G and a streptavidin binding peptide: GS-TAP tag) was constructed. PCR was performed using the *dhtt* “mini-gene” comprising the *dhtt* full length cDNA with intron 10 (as described in [13]) and the following primers: HTT-TAP-FOR: ggtaccATGGACAAATCCAGGTCCAG (KpnI site added) and HTT-TAP-REV: tctagaCAGGCACTGCAACATCCGG (XbaI site added). The resulting PCR product was digested with KpnI/XbaI and sublconed into the *pUAST-CTAP(SG)* vector [54]. To avoid rearrangements due to *dhtt* instability, culturing conditions were used as previously described [13]. *pUAS-htt-fl-CTAP* transgenic flies were generated and balanced using standard procedures and expression of dhtt-SG was assessed using western blots.

### FRET imaging

Fly brains were dissected in 1X PBS at room temperature and collected in ice-cold PBS before being fixed in 3.6% formaldehyde for 20 min. Brains were rinsed twice in PBST 0.5X for 20 min before being mounted in Vectashield (Vector Laboratories). MBs were imaged on adjacent 0.5 μm confocal planes along the anterior-posterior axis using a LSM780 confocal microscope (Zeiss) at x40 with oil immersion. Cyan fluorescent protein (CFP) was excited at 405 nm and emission recorded between 454 and 500 nm. Yellow fluorescent protein (YFP) was excited at 514 nm and emission recorded between 516 and 571 nm. Fluorescence resonance energy transfer (FRET) was generated at 405 nm. To avoid CFP emission, FRET was recorded out of CFP emission range, between 587 and 624 nm.

### FRET image analysis

Brains were oriented anterior-posteriorly using the peduncle as an anatomical landmark and aligned according to the first confocal plane where a signal was visible. We ensured that the same number of planes were obtained for each group (*c739>Abl-FRET*: 48 ± 4 planes and *c739>(Y➔F) Abl-FRET*: 46 ± 1.5 planes, Student t-test: P = 0,7. *c739>Abl-FRET*: 79,3 ± 2,3 planes and *c739; htt*^*int*^*>Abl-FRET*: 75,4 ± 3,7 planes; Student t-test: P = 0,4) indicating that there were no differences due to mounting. Average YFP and FRET signals were computed using the measurement of ‘Mean Grey Value’ and the ‘Plot Z-axis Profile’ functions of ImageJ [55] into a region of interest (ROI) corresponding to the contour of the MB and for each confocal plane. Background was corrected using the ‘Rolling Ball Background Subtraction’ function (50 px radius). For each plane and within the same ROI, FRET signal was expressed relative to YFP to account for variability in Abl-FRET biosensor expression level or differences between preparations. Only groups (i.e. *Abl-FRET* vs *(Y➔F) Abl-FRET* and *Abl-FRET* vs *htt*^*int*^*;Abl-FRET*) crossed, collected, dissected and imaged on the same day were compared. For any given confocal plane, the FRET ratio was averaged between left and right MBs and multiple genetically identical animals.

### qRT-PCR

To quantify *Abl* expression, RNA was extracted from the brains of L3 males. Brains (~20/sample) were dissected in PBS 1X (Sigma) and kept on ice before homogenized in Trizol reagent (Ambion). Total RNA was treated with DNAse to eliminate genomic DNA (Applied Biosystems). RNA was purified using phenol-chloroform extraction and first strand cDNA synthesis was performed using reverse transcriptase (Invitrogen). Primers for *Abl* RNA amplification were designed on each side of intron 4–5 within exon 4 and 5 respectively. These exons are present in all *Abl* transcripts. *Abl* primers were designed using Primer3Plus online software [56]. *Abl* forward primer sequence is 5’-GCGGCCATCATGAAGGAAATG-3’ and reverse primer sequence is 5’-TTGCCGTGCGACATAAACTC-3’. *Abl* RNAs were quantified in real-time during amplification using incorporation of SYBRGreen (Roche) and Light Cycler (Roche). Primers efficacy was first evaluated using a range of cDNA concentrations to ensure linearity of the amplification (E = 1,944). Only a single PCR product with the expected melting temperature was obtained. The amplicon was run on a gel to verify that the size was as expected for the spliced *Abl* product (107 bp). A control without reverse transcription was done to ensure that *Abl* amplicon was not obtained. For each sample, a technical triplicate was performed and averaged. Independent biological replicates were prepared for each condition and the fold change was averaged (see Statistics). The biological replicates correspond to independent dissections, extractions, reverse transcriptions and quantifications. In the experimental condition (*y w*^*67c23*^/Y;; *htt*^*int*^/+), *Abl* expression was expressed relative to control flies (*y w*^*67c23*^/Y) after normalization to internal controls, *Rpl9* and *Rpl32*, and using the ΔΔCT method [57].

### Western blotting

Lysates of adult *Drosophila* heads were prepared using RIPA buffer supplemented with protease inhibitors (Sigma #11836170001). Antibodies used for immunoblotting with dilutions were: anti-dhtt (3526, rabbit polyclonal, 1:1,000; [13]), anti-dAbl (as above, 1:1,000; [58]), anti-β-Tubulin (mouse, DSHB E7, 1:10,000), anti-mouse and anti-rabbit IRDye secondary antibodies (LI-COR Biosciences, 1:10,000). Lysates were resolved on NuPAGE 3–8% gradient Tris-Acetate gels with Tris-Acetate running buffer (for Htt and Abl) or NuPAGE 4–12% gradient Bis-Tris gels with MOPS running buffer (for β-Tubulin). After transfer to nitrocellulose membranes, blots were processed according to the Odyssey CLx protocol. Median band intensity was quantified using Image Studio.

### Statistics

Comparisons between two groups expressing a qualitative variable were analyzed for statistical significance using the Chi^2^ or the Fisher exact test (BiostaTGV: http://biostatgv.sentiweb.fr/?module=tests). Comparison of two groups expressing a quantitative variable was analyzed using the two-tailed Mann-Whitney *U* test or the unpaired *t*-test. For FRET quantitation, statistical analyses were performed using Prism 8.0 (GraphPad). For each confocal plane of each group, the normality of the FRET ratio was assessed using D’Agostino & Pearson normality test. Non-parametric Mann-Whitney tests were used to compare groups at each confocal plane. For the RT-qPCR, the averaged fold change of *Abl* expression was compared to the theoretical value of 1 that would correspond to no change in *Abl* expression and non-parametric Wilcoxon signed-rank test for non-normally distributed data or small samples was used. For Western blots, significance was quantified using Prism 8.0 (GraphPad) and calculated by one-way ANOVA (Figs 6E, 6F and S2) or by unpaired Student’s *t*-test (Fig 6H and 6I). Values of P < 0.05 were considered to be significant.

Detailed R commands for the statistical analysis of Figs 1E and 4D are accessible at: [https://github.com/HKeyHKey/Marquilly_et_al_2020/blob/master/README.md]

## Supporting information

S1 Fig The overexpression of *Abl* rescues the *Appl*^*d*^ and the *dsh*^*1*^ mutant phenotypes. (A) The loss of *htt* does not produce *per se* any significant MB developmental defects. (B) Quantitation of the rescue of *Appl*^*d*^ MB phenotype by the genomic construct *Abl-GFP*. (C) Quantitation of the rescue of *dsh*^*1*^ phenotype by the *Abl-GFP* genomic construct. n = number of MBs analyzed, * P < 0.05 and *** P < 0.001 (Chi^2^ test). All panels correspond to adult brains. Genotypes: (A) top to bottom: *y w*^*67c23*^ / Y*; c739-GAL4 UAS-mito-GFP / +*; *htt-ko* / +. *y w*^*67c23*^ / Y*; c739-GAL4 UAS-mito-GFP / +*; *Df-htt* / +. *y w*^*67c23*^ / Y*;*; *Df-htt / htt-ko*. *y w*^*67c23*^ / Y*;*; *htt*^*int*^ */ +*. *y w*^*67c23*^ / Y;; *htt*^*int*^ */ htt*^*int*^. *y w*^*67c23*^ / Y*; c739-GAL4 UAS-mito-GFP / UAS-RNAi-htt*. *y w*^*67c23*^/ Y*; UAS-mCD8-GFP / UAS-RNAi-htt;; OK107-GAL4 / +*. (B) top to bottom: *Appl*^*d*^ w* / Y*; c739-GAL4 UAS-mito-GFP / +*. *Appl*^*d*^ w* / Y*; c739-GAL4 UAS-mito-GFP / Abl-GFP*. (C) top to bottom: *w dsh*^*1*^ / Y. *w dsh*^*1*^ / Y; *Abl-GFP* / +.
(TIF)

S2 Fig Structure and overexpression of the Abl protein. (A) Molecular scheme of the Abl protein. Abl protein is composed of conserved domains: Src Homology 3 (SH3) domain (blue), Src Homology 2 (SH2) domain (orange), Kinase Domain (red), Poly-Proline PP domain (purple) and F-Actin Binding Domain (FABD) (green). The protein produced by *Abl*^*1*^ mutant allele is truncated between PP and Kinase domains. The protein produced by *Abl*^*2*^ mutant allele is truncated within the Kinase domain. The protein produced by *Abl*^*4*^ mutant allele is truncated within the SH2 domain [47]. (B-E) Anti-Fas2 staining showing the α and β lobes in a WT adult brain (B) and in *Abl* forced expression by *OK107-GAL4* (C-E) with an absence of β lobe (C), an absence of α lobe (D) and an absence of α and β lobes (E). The loss of lobes is emphasized by white dashed lines. Note that panel B is also presented as the left MB in Fig 1A. The scale bar indicates 30 μm. Images are composite stacks. Genotypes: (B) *y w*^*67c23*^ / Y. (C-E) *y w*^*67c23*^ / Y; *UAS-Abl / UAS-mCD8-GFP;; OK107-GAL4 /* +. (F) The expression levels of *UAS-Abl* and *UAS-Abl*^*KD*^ transgenes are similar. *UAS-Abl* and *UAS-Abl*^*KD*^ were expressed using *OK107-GAL4*. Lysates from adult heads were subjected to western blotting to assess Abl and β-Tubulin (β-Tub) levels. 1, 2, and 3 indicate biological replicates for each line. (G) Quantitation of Abl protein levels in the indicated genotypes was assessed from western blots in (F) relative to β-Tubulin and plotted as relative band intensity. Errors indicate standard deviation. Significance was calculated by one-way ANOVA (P < 0.0001) followed by post-hoc Bonferroni’s multiple comparison correction (n.s. not statistically different and **** P < 0.0001). (H-L”‘) GFP (green) labelling is showing all the lobes (α and α’ vertically, β and β’ and γ medially), anti-Trio (red) staining is showing the α’ and β’ and γ lobes and anti-Fas2 (blue) staining is showing the α and β and weakly the γ lobes in a WT adult brain (H-H”‘) and in *Abl* forced expression by *OK107-GAL4* (I-L”‘) with an absence of β’ lobe (I-I”‘), an absence of α’ lobe (J-J”‘) and an absence of α’ and β’ lobes (K-K”‘). In H’ and H”‘ the α’ lobe, indicated by a yellow arrowhead, projects vertically and the β’ lobe, indicated by a pink arrowhead, projects toward the midline. In I’, I”‘ and J’, J”‘ the present lobes are indicated by arrowheads although the absent lobes are indicated by empty arrowheads. (L-L”‘) is a single confocal section from K-K”‘. The loss of lobes is emphasized by white dashed lines. The scale bar indicates 30 μm. Images are composite stacks. Genotypes: (H-H”‘) *y w*^*67c23*^/ Y; + */ UAS-mCD8-GFP;; OK107-GAL4 /* +. (I-L”‘) *y w*^*67c23*^ / Y; *UAS-Abl / UAS-mCD8-GFP;; OK107-GAL4 /* +.
(TIF)

S3 Fig *Abl* loss of function in MBs. (A-A’) Neuroblast WT αβ neuron MARCM clone in a WT brain (A) associated with anti-Fas2 staining in red (A’). (B-B’) Neuroblast WT-looking αβ neuron clone (B) associated with anti- Fas2 staining in red (B’) in an *Abl*^*2*^*/Abl*^*4*^ brain. (C-C’) Neuroblast αβ neuron clone with an absence of α branch (C) associated with anti-Fas2 staining in red (C’) in an *Abl*^*2*^*/Abl*^*4*^ brain displaying an absence of α lobe. (D-D*’*) Neuroblast αβ neuron clone with shorter α and β branches (D) associated with anti-Fas2 staining in red (D’) in an *Abl*^*2*^*/Abl*^*4*^ brain displaying an absence of α and β lobes. (E-E’) Multicell WT αβ neuron MARCM clone in a WT brain (E) associated with anti-Fas2 staining in red (E’). (F-F’) Multicell WT-looking αβ neuron clone (F) associated with anti-Fas2 staining in red (F’) in an *Abl*^*2*^*/Abl*^*4*^ brain. (G-G’) Multicell αβ neuron clone with an absence of α branches (G) associated with anti-Fas2 staining in red (G’) in an *Abl*^*2*^*/Abl*^*4*^ brain displaying an absence of α lobe. (H-H*’*) Multicell αβ neuron clone with shorter α and β branches (H) associated with anti-Fas2 staining in red (H’) in an *Abl*^*2*^*/Abl*^*4*^ brain displaying an absence of α and β lobes. (A-H’) All panels correspond to 48 hAPF brains. (I-L) Anti-Fas2 staining on wild-type (WT) brain (I) and on *Abl*^*2*^*/Abl*^*4*^ brain (J-L) at L3 larval stage. In a wild-type (WT) brain, γ neurons project to vertical and medial lobes. In an *Abl*^*2*^*/Abl*^*4*^ brain, 60% of γ neurons are WT (J) whereas, 27% show a loss of the medial lobe (K) and 13% show a loss of both vertical and medial lobes (L). The loss of the vertical and medial lobes is emphasized by white dashed lines. The scale bar in panels A-L indicates 10 μm. Images are composite stacks to allow the visualization of axon trajectories along their entire length. Genotypes: (A and E) w* tubP-GAL80 hs-FLP122 FRT19A / w* sn FRT19A; c739-GAL4 UAS-mCD8-GFP / UAS-mCD8-GFP. (B-D and F-H) w* tubP-GAL80 hs-FLP122 FRT19A / w* sn FRT19A; c739-GAL4 UAS-mCD8-GFP / UAS-mCD8-GFP; Abl^*2*^ */ Abl*^*4*^. (I) *y w*^*67c23*^. (J-L) *y w*^*67c23*^*;; Abl*^*2*^ *FRT2A / Abl*^*4*^ *FRT2A*.
(TIF)

S4 Fig *htt* interaction with *Abl*. The loss of one copy of *htt* does not rescue the *Abl*^*4*^/*Abl*^*1*^ (A) or *Abl*^*2*^/*Abl*^*4*^ (B) mutant phenotype. All panels correspond 48 hAPF brains. n = number of MBs analyzed with P = 0.22 for *Abl*^*4*^/*Abl*^*1*^ and P = 0.70 for *Abl*^*2*^/*Abl*^*4*^ (Fisher exact test). Genotypes: top to bottom: *y w*^*67c23*^;; *Abl*^*4*^ *FRT2A / Abl*^*1*^ *FRT2A*. *y w*^*67c23*^*;; Abl*^*4*^ *FRT2A htt*^*int*^ */ Abl*^*1*^ *FRT2A*. *y w*^*67c23*^*;; Abl*^*2*^ *FRT2A / Abl*^*4*^ *FRT2A*. *y w*^*67c23*^*;; Abl*^*2*^ *FRT2A htt*^*int*^ */ Abl*^*4*^ *FRT2A*.
(TIF)

S5 Fig Detection of the Abl-FRET biosensor in the MBs. (*top*) Maximum intensity projection of α and β MB lobes in adult flies. (*bottom*) Maximum intensity projection of vertical and medial MB lobes in stage 3 larvae. The Abl-FRET biosensor is expressed in the MBs using *c739-GAL4* and imaged. Maximum intensity projection of confocal stacks corresponding to YFP and FRET signal are shown. These are presented as examples of the kind of image data that goes into the FRET ratio calculation. Scale bar: 10μm.
(TIF)

## References

[1] Soto C, Pritzkow S. Protein misfolding, aggregation, and conformational strains in neurodegenerative diseases. Nat Neurosci. 2018;21(10):1332–40. Epub 2018/09/27. 10.1038/s41593-018-0235-9 30250260 PMC6432913

[2] Dugger BN, Dickson DW. Pathology of Neurodegenerative Diseases. Cold Spring Harb Perspect Biol. 2017;9(7). Epub 2017/01/08. 10.1101/cshperspect.a028035 . 28062563 PMC5495060

[3] Arneson D, Zhang Y, Yang X, Narayanan M. Shared mechanisms among neurodegenerative diseases: from genetic factors to gene networks. J Genet. 2018;97(3):795–806. Epub 2018/07/22. . 30027910 PMC6211183

[4] Cattaneo E, Zuccato C, Tartari M. Normal huntingtin function: an alternative approach to Huntington’s disease. Nat Rev Neurosci. 2005;6(12):919–30. Epub 2005/11/17. 10.1038/nrn1806 . 16288298

[5] Soldano A, Okray Z, Janovska P, Tmejova K, Reynaud E, Claeys A, et al The Drosophila Homologue of the Amyloid Precursor Protein Is a Conserved Modulator of Wnt PCP Signaling. PLoS biology. 2013;11(5):e1001562 Epub 2013/05/22. 10.1371/journal.pbio.1001562 . 23690751 PMC3653798

[6] Serra HG, Duvick L, Zu T, Carlson K, Stevens S, Jorgensen N, et al RORalpha-mediated Purkinje cell development determines disease severity in adult SCA1 mice. Cell. 2006;127(4):697–708. Epub 2006/11/18. 10.1016/j.cell.2006.09.036 . 17110330

[7] Bothwell M, Giniger E. Alzheimer’s disease: neurodevelopment converges with neurodegeneration. Cell. 2000;102(3):271–3. Epub 2000/09/07. 10.1016/s0092-8674(00)00032-5 . 10975517

[8] Barnat M, Capizzi M, Aparicio E, Boluda S, Wennagel D, Kacher R, et al Huntington’s disease alters human neurodevelopment. Science. 2020 Epub 2020/07/18. 10.1126/science.aax3338 . 32675289 PMC7859879

[9] Saudou F, Humbert S. The Biology of Huntingtin. Neuron. 2016;89(5):910–26. Epub 2016/03/05. 10.1016/j.neuron.2016.02.003 . 26938440

[10] Arribat Y, Bonneaud N, Talmat-Amar Y, Layalle S, Parmentier ML, Maschat F. A huntingtin peptide inhibits polyQ-huntingtin associated defects. PLoS One. 2013;8(7):e68775 Epub 2013/07/19. 10.1371/journal.pone.0068775 . 23861941 PMC3701666

[11] Pouladi MA, Morton AJ, Hayden MR. Choosing an animal model for the study of Huntington’s disease. Nat Rev Neurosci. 2013;14(10):708–21. Epub 2013/09/21. 10.1038/nrn3570 . 24052178

[12] Zhang S, Feany MB, Saraswati S, Littleton JT, Perrimon N. Inactivation of Drosophila Huntingtin affects long-term adult functioning and the pathogenesis of a Huntington’s disease model. Dis Model Mech. 2009;2(5–6):247–66. Epub 2009/04/22. 10.1242/dmm.000653 . 19380309 PMC2675792

[13] Dietz KN, Di Stefano L, Maher RC, Zhu H, Macdonald ME, Gusella JF, et al The Drosophila Huntington’s disease gene ortholog dhtt influences chromatin regulation during development. Hum Mol Genet. 2015;24(2):330–45. Epub 2014/08/30. 10.1093/hmg/ddu446 . 25168387

[14] Kunkle BW, Grenier-Boley B, Sims R, Bis JC, Damotte V, Naj AC, et al Author Correction: Genetic meta-analysis of diagnosed Alzheimer’s disease identifies new risk loci and implicates Abeta, tau, immunity and lipid processing. Nat Genet. 2019;51(9):1423–4. Epub 2019/08/17. 10.1038/s41588-019-0495-7 . 31417202 PMC7265117

[15] Kunkle BW, Grenier-Boley B, Sims R, Bis JC, Damotte V, Naj AC, et al Genetic meta-analysis of diagnosed Alzheimer’s disease identifies new risk loci and implicates Abeta, tau, immunity and lipid processing. Nat Genet. 2019;51(3):414–30. Epub 2019/03/02. 10.1038/s41588-019-0358-2 . 30820047 PMC6463297

[16] Bellenguez C, Grenier-Boley B, Lambert JC. Genetics of Alzheimer’s disease: where we are, and where we are going. Curr Opin Neurobiol. 2020;61:40–8. Epub 2019/12/22. 10.1016/j.conb.2019.11.024 . 31863938

[17] Mlodzik M. The Dishevelled Protein Family: Still Rather a Mystery After Over 20 Years of Molecular Studies. Curr Top Dev Biol. 2016;117:75–91. Epub 2016/03/13. 10.1016/bs.ctdb.2015.11.027 . 26969973 PMC4939608

[18] Sharma M, Castro-Piedras I, Simmons GE Jr., Pruitt K. Dishevelled: A masterful conductor of complex Wnt signals. Cell Signal. 2018;47:52–64. Epub 2018/03/22. 10.1016/j.cellsig.2018.03.004 . 29559363 PMC6317740

[19] Singh J, Yanfeng WA, Grumolato L, Aaronson SA, Mlodzik M. Abelson family kinases regulate Frizzled planar cell polarity signaling via Dsh phosphorylation. Genes Dev. 2010;24(19):2157–68. Epub 2010/09/15. 10.1101/gad.1961010 . 20837657 PMC2947768

[20] Colicelli J. ABL tyrosine kinases: Evolution of function, regulation, and specificity. Sci Signal. 2010;3(139):re6 10.1126/scisignal.3139re6 20841568 PMC2954126

[21] Wang JYJ. The capable ABL: what is its biological function? Mol Cell Biol. 2014;34(7). 10.1128/MCB.01454-13 24421390 PMC3993570

[22] Barila D, Superti-Furga G. An intramolecular SH3-domain interaction regulates c-Abl activity. Nat Genet. 1998;18(3):280–2. Epub 1998/03/21. 10.1038/ng0398-280 . 9500553

[23] Wen ST, Van Etten RA. The PAG gene product, a stress-induced protein with antioxidant properties, is an Abl SH3-binding protein and a physiological inhibitor of c-Abl tyrosine kinase activity. Genes Dev. 1997;11(19):2456–67. Epub 1997/10/23. 10.1101/gad.11.19.2456 . 9334312 PMC316562

[24] Brasher BB, Van Etten RA. c-Abl has high intrinsic tyrosine kinase activity that is stimulated by mutation of the Src homology 3 domain and by autophosphorylation at two distinct regulatory tyrosines. J Biol Chem. 2000;275(45):35631–7. Epub 2000/08/31. . 10964922 10.1074/jbc.M005401200

[25] Schlatterer SD, Acker CM, Davies P. c-Abl in neurodegenerative disease. J Mol Neurosci. 2011;45(3):445–52. Epub 2011/07/06. 10.1007/s12031-011-9588-1 . 21728062 PMC3329755

[26] Fogerty FJ, Juang JL, Petersen J, Clark MJ, Hoffmann FM, Mosher DF. Dominant effects of the bcr-abl oncogene on Drosophila morphogenesis. Oncogene. 1999;18(1):219–32. Epub 1999/02/02. 10.1038/sj.onc.1202239 . 9926937

[27] Li W, Li Y, Gao FB. Abelson, enabled, and p120 catenin exert distinct effects on dendritic morphogenesis in Drosophila. Dev Dyn. 2005;234(3):512–22. Epub 2005/07/09. 10.1002/dvdy.20496 . 16003769

[28] Xiong W, Rebay I. Abelson tyrosine kinase is required for Drosophila photoreceptor morphogenesis and retinal epithelial patterning. Dev Dyn. 2011;240(7):1745–55. Epub 2011/06/16. 10.1002/dvdy.22674 . 21674685 PMC3314402

[29] Wills Z, Bateman J, Korey CA, Comer A, Van Vactor D. The tyrosine kinase Abl and its substrate enabled collaborate with the receptor phosphatase Dlar to control motor axon guidance. Neuron. 1999;22(2):301–12. Epub 1999/03/09. 10.1016/s0896-6273(00)81091-0 . 10069336

[30] Wills Z, Marr L, Zinn K, Goodman CS, Van Vactor D. Profilin and the Abl tyrosine kinase are required for motor axon outgrowth in the Drosophila embryo. Neuron. 1999;22(2):291–9. Epub 1999/03/09. 10.1016/s0896-6273(00)81090-9 . 10069335

[31] Crowner D, Le Gall M, Gates MA, Giniger E. Notch steers Drosophila ISNb motor axons by regulating the Abl signaling pathway. Curr Biol. 2003;13(11):967–72. Epub 2003/06/05. 10.1016/s0960-9822(03)00325-7 . 12781136

[32] Clarke A, McQueen PG, Fang HY, Kannan R, Wang V, McCreedy E, et al Dynamic morphogenesis of a pioneer axon in Drosophila and its regulation by Abl tyrosine kinase. Mol Biol Cell. 2020;31(6):452–65. Epub 2020/01/23. 10.1091/mbc.E19-10-0563 . 31967935 PMC7185889

[33] Clarke A, McQueen PG, Fang HY, Kannan R, Wang V, McCreedy E, et al Abl signaling directs growth of a pioneer axon in Drosophila by shaping the intrinsic fluctuations of actin. Mol Biol Cell. 2020;31(6):466–77. Epub 2020/01/23. 10.1091/mbc.E19-10-0564 . 31967946 PMC7185895

[34] Leyssen M, Ayaz D, Hebert SS, Reeve S, De Strooper B, Hassan BA. Amyloid precursor protein promotes post-developmental neurite arborization in the Drosophila brain. EMBO J. 2005;24(16):2944–55. Epub 2005/07/30. 10.1038/sj.emboj.7600757 . 16052209 PMC1187942

[35] Busto GU, Cervantes-Sandoval I, Davis RL. Olfactory learning in Drosophila. Physiology (Bethesda). 2010;25(6):338–46. Epub 2010/12/28. 10.1152/physiol.00026.2010 . 21186278 PMC3380424

[36] Heisenberg M. Mushroom body memoir: from maps to models. Nat Rev Neurosci. 2003;4(4):266–75. 10.1038/nrn1074 . 12671643

[37] Lee T, Lee A, Luo L. Development of the Drosophila mushroom bodies: sequential generation of three distinct types of neurons from a neuroblast. Development. 1999;126(18):4065–76. . 10457015 10.1242/dev.126.18.4065

[38] Ng J. Wnt/PCP proteins regulate stereotyped axon branch extension in Drosophila. Development. 2012;139(1):165–77. Epub 2011/12/08. 10.1242/dev.068668 . 22147954 PMC3231775

[39] Luo L, Tully T, White K. Human amyloid precursor protein ameliorates behavioral deficit of flies deleted for Appl gene. Neuron. 1992;9(4):595–605. Epub 1992/10/01. 10.1016/0896-6273(92)90024-8 . 1389179

[40] Reynaud E, Lahaye LL, Boulanger A, Petrova IM, Marquilly C, Flandre A, et al Guidance of Drosophila Mushroom Body Axons Depends upon DRL-Wnt Receptor Cleavage in the Brain Dorsomedial Lineage Precursors. Cell Rep. 2015;11(8):1293–304. Epub 2015/05/20. 10.1016/j.celrep.2015.04.035 . 25981040

[41] Brand AH, Perrimon N. Targeted gene expression as a means of altering cell fates and generating dominant phenotypes. Development. 1993;118(2):401–15. 8223268 10.1242/dev.118.2.401

[42] Kannan R, Song JK, Karpova T, Clarke A, Shivalkar M, Wang B, et al The Abl pathway bifurcates to balance Enabled and Rac signaling in axon patterning in Drosophila. Development. 2017;144(3):487–98. Epub 2017/01/15. 10.1242/dev.143776 . 28087633 PMC5341800

[43] Staropoli JF. Tumorigenesis and neurodegeneration: two sides of the same coin? Bioessays. 2008;30(8):719–27. Epub 2008/07/16. 10.1002/bies.20784 . 18623069

[44] Thion MS, Humbert S. Cancer: From Wild-Type to Mutant Huntingtin. J Huntingtons Dis. 2018;7(3):201–8. Epub 2018/06/12. 10.3233/JHD-180290 . 29889077 PMC6087435

[45] Bowles KR, Jones L. Kinase signalling in Huntington’s disease. J Huntingtons Dis. 2014;3(2):89–123. Epub 2014/07/27. 10.3233/JHD-140106 . 25062854

[46] Beckett C, Nalivaeva NN, Belyaev ND, Turner AJ. Nuclear signalling by membrane protein intracellular domains: the AICD enigma. Cell Signal. 2012;24(2):402–9. Epub 2011/10/26. 10.1016/j.cellsig.2011.10.007 . 22024280

[47] Smith JA, Liebl EC. Identification of the molecular lesions in alleles of the Drosophila Abelson tyrosine kinase. Dros Inf Serv. 2005;88:20–2.

[48] Bennett RL, Hoffmann FM. Increased levels of the Drosophila Abelson tyrosine kinase in nerves and muscles: subcellular localization and mutant phenotypes imply a role in cell-cell interactions. Development. 1992;116(4):953–66. Epub 1992/12/01. . 1295746 10.1242/dev.116.4.953

[49] Gunawardena S, Her LS, Brusch RG, Laymon RA, Niesman IR, Gordesky-Gold B, et al Disruption of axonal transport by loss of huntingtin or expression of pathogenic polyQ proteins in Drosophila. Neuron. 2003;40(1):25–40. Epub 2003/10/07. 10.1016/s0896-6273(03)00594-4 . 14527431

[50] Fox DT, Peifer M. Abelson kinase (Abl) and RhoGEF2 regulate actin organization during cell constriction in Drosophila. Development. 2007;134(3):567–78. Epub 2007/01/05. 10.1242/dev.02748 . 17202187

[51] Aso Y, Grubel K, Busch S, Friedrich AB, Siwanowicz I, Tanimoto H. The mushroom body of adult Drosophila characterized by GAL4 drivers. J Neurogenet. 2009;23(1–2):156–72. Epub 2009/01/14. 10.1080/01677060802471718 . 19140035

[52] Boulanger A, Clouet-Redt C, Farge M, Flandre A, Guignard T, Fernando C, et al ftz-f1 and Hr39 opposing roles on EcR expression during Drosophila mushroom body neuron remodeling. Nat Neurosci. 2011;14(1):37–44. Epub 2010/12/07. 10.1038/nn.2700 . 21131955

[53] Lee T, Luo L. Mosaic analysis with a repressible cell marker for studies of gene function in neuronal morphogenesis. Neuron. 1999;22(3):451–61. 10.1016/s0896-6273(00)80701-1 . 10197526

[54] Kyriakakis P, Tipping M, Abed L, Veraksa A. Tandem affinity purification in Drosophila: the advantages of the GS-TAP system. Fly (Austin). 2008;2(4):229–35. Epub 2008/08/23. 10.4161/fly.6669 . 18719405

[55] Schneider CA, Rasband WS, Eliceiri KW. NIH Image to ImageJ: 25 years of image analysis. Nat Methods. 2012;9(7):671–5. Epub 2012/08/30. 10.1038/nmeth.2089 . 22930834 PMC5554542

[56] Untergasser A, Cutcutache I, Koressaar T, Ye J, Faircloth BC, Remm M, et al Primer3—new capabilities and interfaces. Nucleic Acids Res. 2012;40(15):e115 Epub 2012/06/26. 10.1093/nar/gks596 . 22730293 PMC3424584

[57] Livak KJ, Schmittgen TD. Analysis of relative gene expression data using real-time quantitative PCR and the 2(-Delta Delta C(T)) Method. Methods. 2001;25(4):402–8. Epub 2002/02/16. 10.1006/meth.2001.1262 . 11846609

[58] Song JK, Kannan R, Merdes G, Singh J, Mlodzik M, Giniger E. Disabled is a bona fide component of the Abl signaling network. Development. 2010;137(21):3719–27. Epub 2010/10/14. 10.1242/dev.050948 . 20940230 PMC2964101

[59] Ting AY, Kain KH, Klemke RL, Tsien RY. Genetically encoded fluorescent reporters of protein tyrosine kinase activities in living cells. Proc Natl Acad Sci U S A. 2001;98(26):15003–8. Epub 2001/12/26. 10.1073/pnas.211564598 . 11752449 PMC64973

