# Supplementary figures and images for: Htt is a repressor of Abl activity required for APP-induced axonal growth
Source: PLoS Genet. 2021 Jan 19;17(1):e1009287. doi: 10.1371/journal.pgen.1009287 (PMC7845969; doi:10.1371/journal.pgen.1009287)

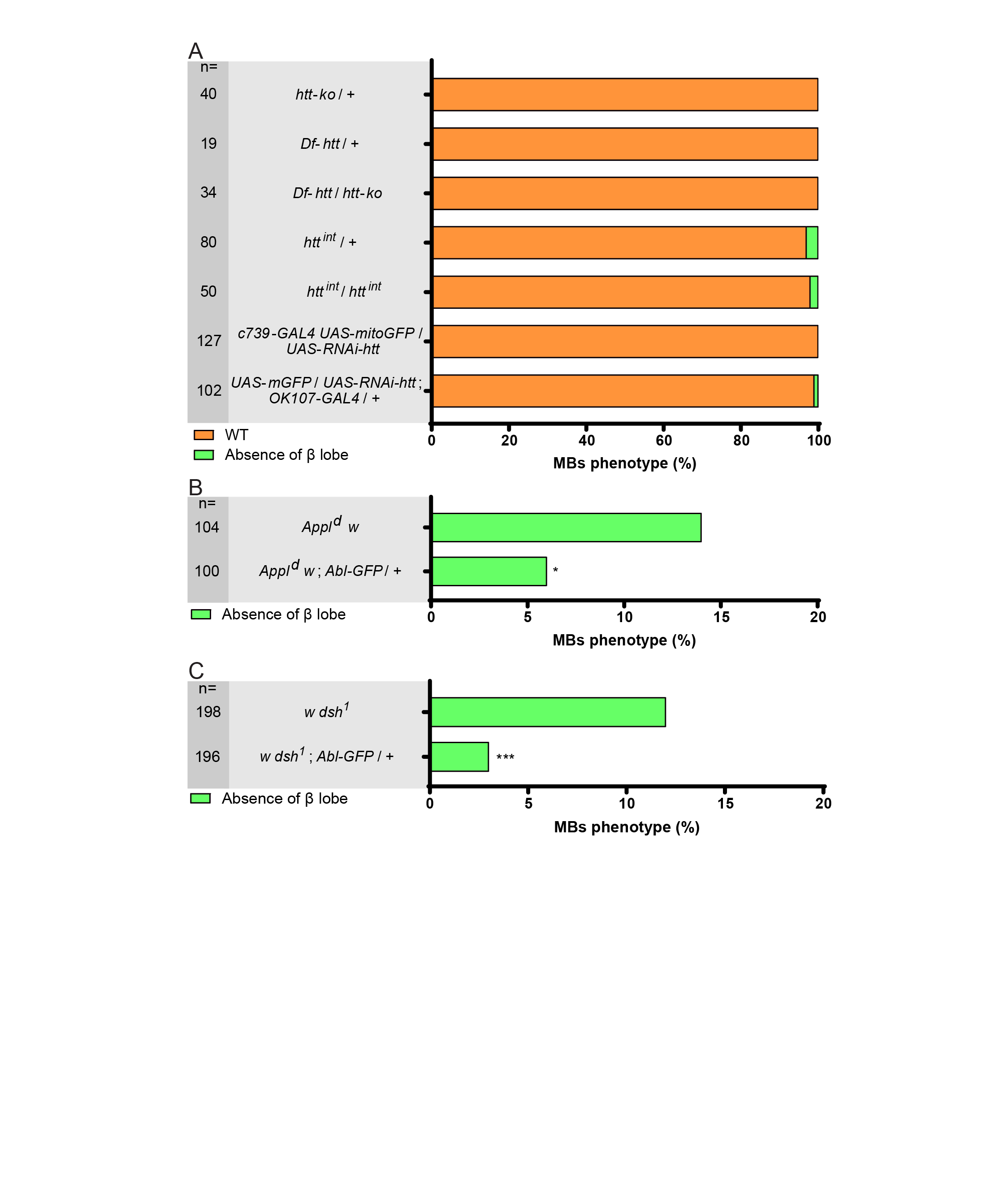

Supplement: S1 Fig — (A) The loss of htt does not produce per se any significant MB developmental defects. (B) Quantitation of the rescue of Appld MB phenotype by the genomic construct Abl-GFP. (C) Quantitation of the rescue of dsh1 phenotype by the Abl-GFP genomic construct. n = number of MBs analyzed, * P < 0.05 and *** P < 0.001 (Chi2 test). All panels correspond to adult brains. Genotypes: (A) top to bottom: y w67c23 / Y; c739-GAL4 UAS-mito-GFP / +; htt-ko / +. y w67c23 / Y; c739-GAL4 UAS-mito-GFP / +; Df-htt / +. y w67c23 / Y;; Df-htt / htt-ko. y w67c23 / Y;; httint / +. y w67c23 / Y;; httint / httint. y w67c23 / Y; c739-GAL4 UAS-mito-GFP / UAS-RNAi-htt. y w67c23/ Y; UAS-mCD8-GFP / UAS-RNAi-htt;; OK107-GAL4 / +. (B) top to bottom: Appld w* / Y; c739-GAL4 UAS-mito-GFP / +. Appld w* / Y; c739-GAL4 UAS-mito-GFP / Abl-GFP. (C) top to bottom: w dsh1 / Y. w dsh1 / Y; Abl-GFP / +. (TIF) [file pgen.1009287.s001.tif]

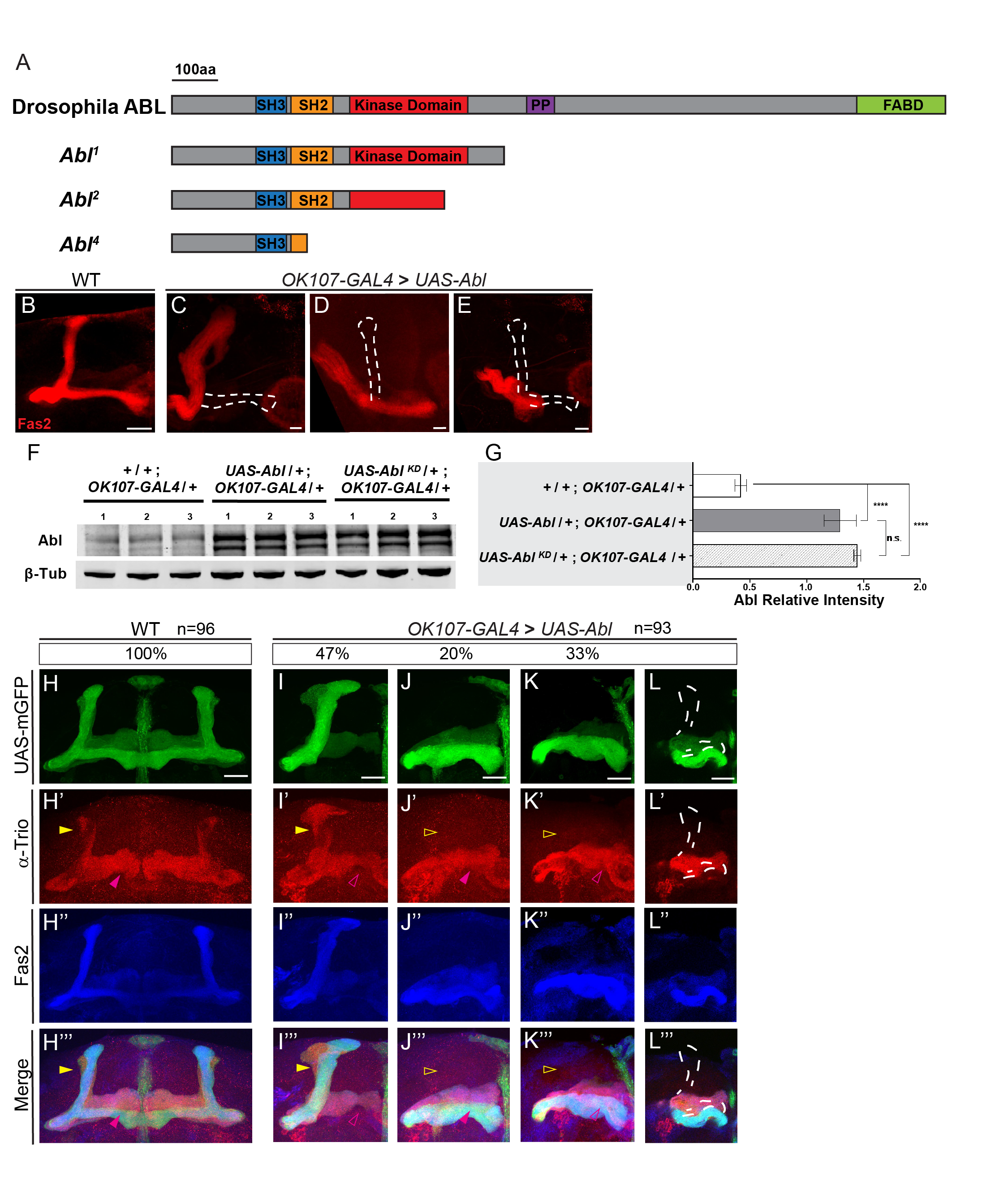

Supplement: S2 Fig — (A) Molecular scheme of the Abl protein. Abl protein is composed of conserved domains: Src Homology 3 (SH3) domain (blue), Src Homology 2 (SH2) domain (orange), Kinase Domain (red), Poly-Proline PP domain (purple) and F-Actin Binding Domain (FABD) (green). The protein produced by Abl1 mutant allele is truncated between PP and Kinase domains. The protein produced by Abl2 mutant allele is truncated within the Kinase domain. The protein produced by Abl4 mutant allele is truncated within the SH2 domain [47]. (B-E) Anti-Fas2 staining showing the α and β lobes in a WT adult brain (B) and in Abl forced expression by OK107-GAL4 (C-E) with an absence of β lobe (C), an absence of α lobe (D) and an absence of α and β lobes (E). The loss of lobes is emphasized by white dashed lines. Note that panel B is also presented as the left MB in Fig 1A. The scale bar indicates 30 μm. Images are composite stacks. Genotypes: (B) y w67c23 / Y. (C-E) y w67c23 / Y; UAS-Abl / UAS-mCD8-GFP;; OK107-GAL4 / +. (F) The expression levels of UAS-Abl and UAS-AblKD transgenes are similar. UAS-Abl and UAS-AblKD were expressed using OK107-GAL4. Lysates from adult heads were subjected to western blotting to assess Abl and β-Tubulin (β-Tub) levels. 1, 2, and 3 indicate biological replicates for each line. (G) Quantitation of Abl protein levels in the indicated genotypes was assessed from western blots in (F) relative to β-Tubulin and plotted as relative band intensity. Errors indicate standard deviation. Significance was calculated by one-way ANOVA (P < 0.0001) followed by post-hoc Bonferroni’s multiple comparison correction (n.s. not statistically different and **** P < 0.0001). (H-L”‘) GFP (green) labelling is showing all the lobes (α and α’ vertically, β and β’ and γ medially), anti-Trio (red) staining is showing the α’ and β’ and γ lobes and anti-Fas2 (blue) staining is showing the α and β and weakly the γ lobes in a WT adult brain (H-H”‘) and in Abl forced expression by OK107-GAL4 (I-L”‘) with an abse [file pgen.1009287.s002.tif]

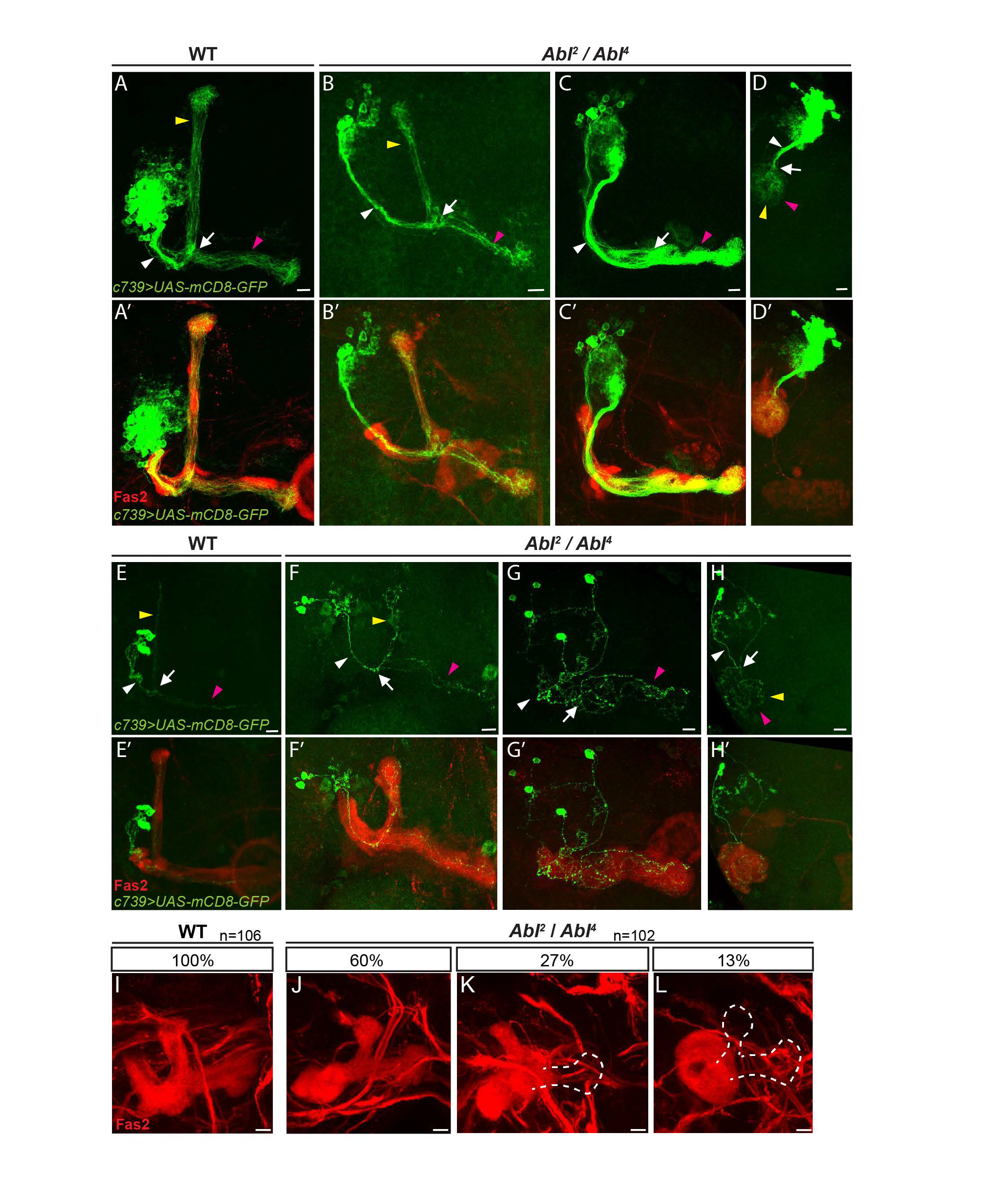

Supplement: S3 Fig — (A-A’) Neuroblast WT αβ neuron MARCM clone in a WT brain (A) associated with anti-Fas2 staining in red (A’). (B-B’) Neuroblast WT-looking αβ neuron clone (B) associated with anti- Fas2 staining in red (B’) in an Abl2/Abl4 brain. (C-C’) Neuroblast αβ neuron clone with an absence of α branch (C) associated with anti-Fas2 staining in red (C’) in an Abl2/Abl4 brain displaying an absence of α lobe. (D-D’) Neuroblast αβ neuron clone with shorter α and β branches (D) associated with anti-Fas2 staining in red (D’) in an Abl2/Abl4 brain displaying an absence of α and β lobes. (E-E’) Multicell WT αβ neuron MARCM clone in a WT brain (E) associated with anti-Fas2 staining in red (E’). (F-F’) Multicell WT-looking αβ neuron clone (F) associated with anti-Fas2 staining in red (F’) in an Abl2/Abl4 brain. (G-G’) Multicell αβ neuron clone with an absence of α branches (G) associated with anti-Fas2 staining in red (G’) in an Abl2/Abl4 brain displaying an absence of α lobe. (H-H’) Multicell αβ neuron clone with shorter α and β branches (H) associated with anti-Fas2 staining in red (H’) in an Abl2/Abl4 brain displaying an absence of α and β lobes. (A-H’) All panels correspond to 48 hAPF brains. (I-L) Anti-Fas2 staining on wild-type (WT) brain (I) and on Abl2/Abl4 brain (J-L) at L3 larval stage. In a wild-type (WT) brain, γ neurons project to vertical and medial lobes. In an Abl2/Abl4 brain, 60% of γ neurons are WT (J) whereas, 27% show a loss of the medial lobe (K) and 13% show a loss of both vertical and medial lobes (L). The loss of the vertical and medial lobes is emphasized by white dashed lines. The scale bar in panels A-L indicates 10 μm. Images are composite stacks to allow the visualization of axon trajectories along their entire length. Genotypes: (A and E) w* tubP-GAL80 hs-FLP122 FRT19A / w* sn FRT19A; c739-GAL4 UAS-mCD8-GFP / UAS-mCD8-GFP. (B-D and F-H) w* tubP-GAL80 hs-FLP122 FRT19A / w* sn FRT19A; c739-GAL4 UAS-mCD8-GFP / UAS-mCD8-GFP; Abl2 / Abl4. (I) y w67c23. (J-L) y w67 [file pgen.1009287.s003.tif]

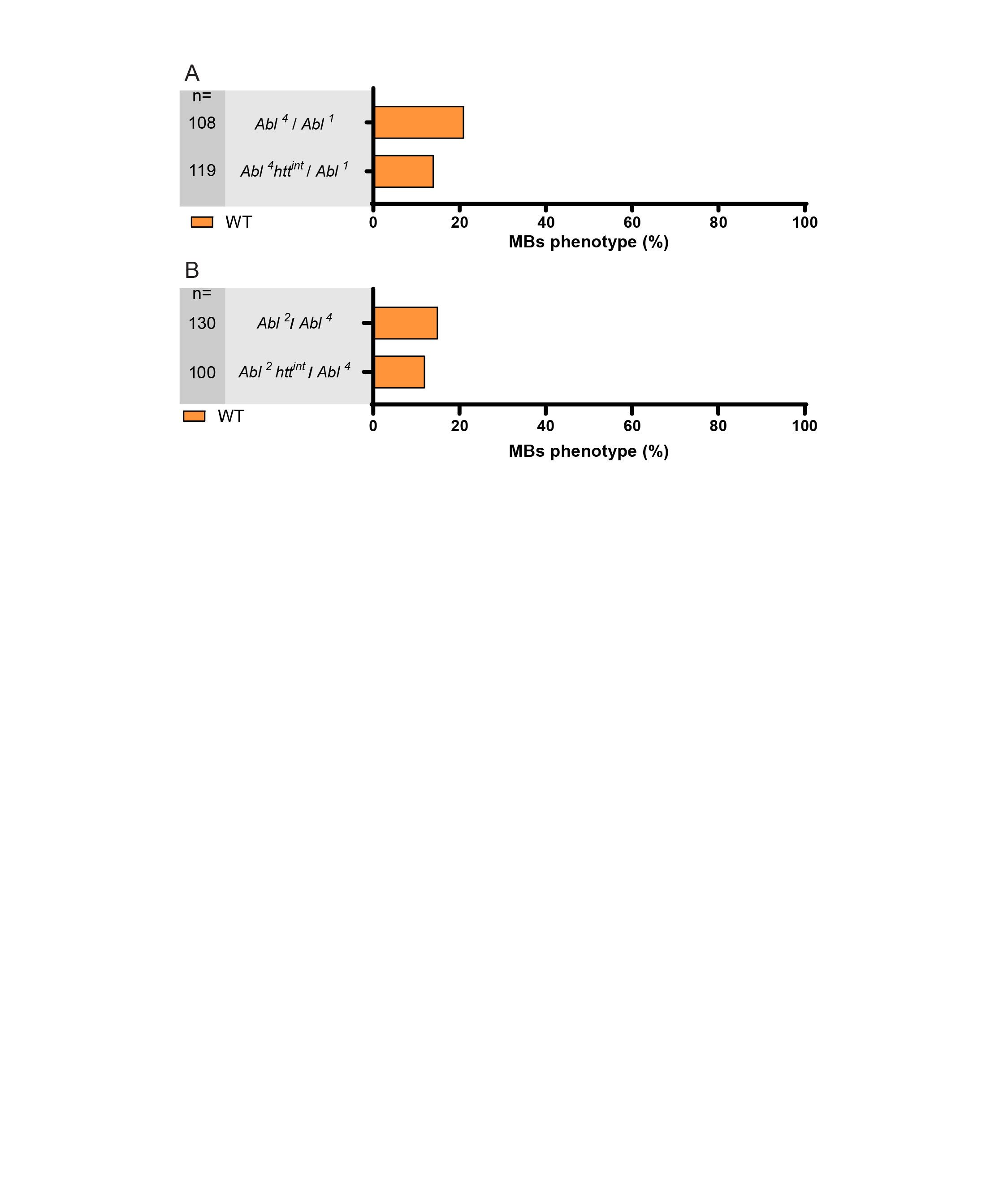

Supplement: S4 Fig — The loss of one copy of htt does not rescue the Abl4/Abl1 (A) or Abl2/Abl4 (B) mutant phenotype. All panels correspond 48 hAPF brains. n = number of MBs analyzed with P = 0.22 for Abl4/Abl1 and P = 0.70 for Abl2/Abl4 (Fisher exact test). Genotypes: top to bottom: y w67c23;; Abl4 FRT2A / Abl1 FRT2A. y w67c23;; Abl4 FRT2A httint / Abl1 FRT2A. y w67c23;; Abl2 FRT2A / Abl4 FRT2A. y w67c23;; Abl2 FRT2A httint / Abl4 FRT2A. (TIF) [file pgen.1009287.s004.tif]

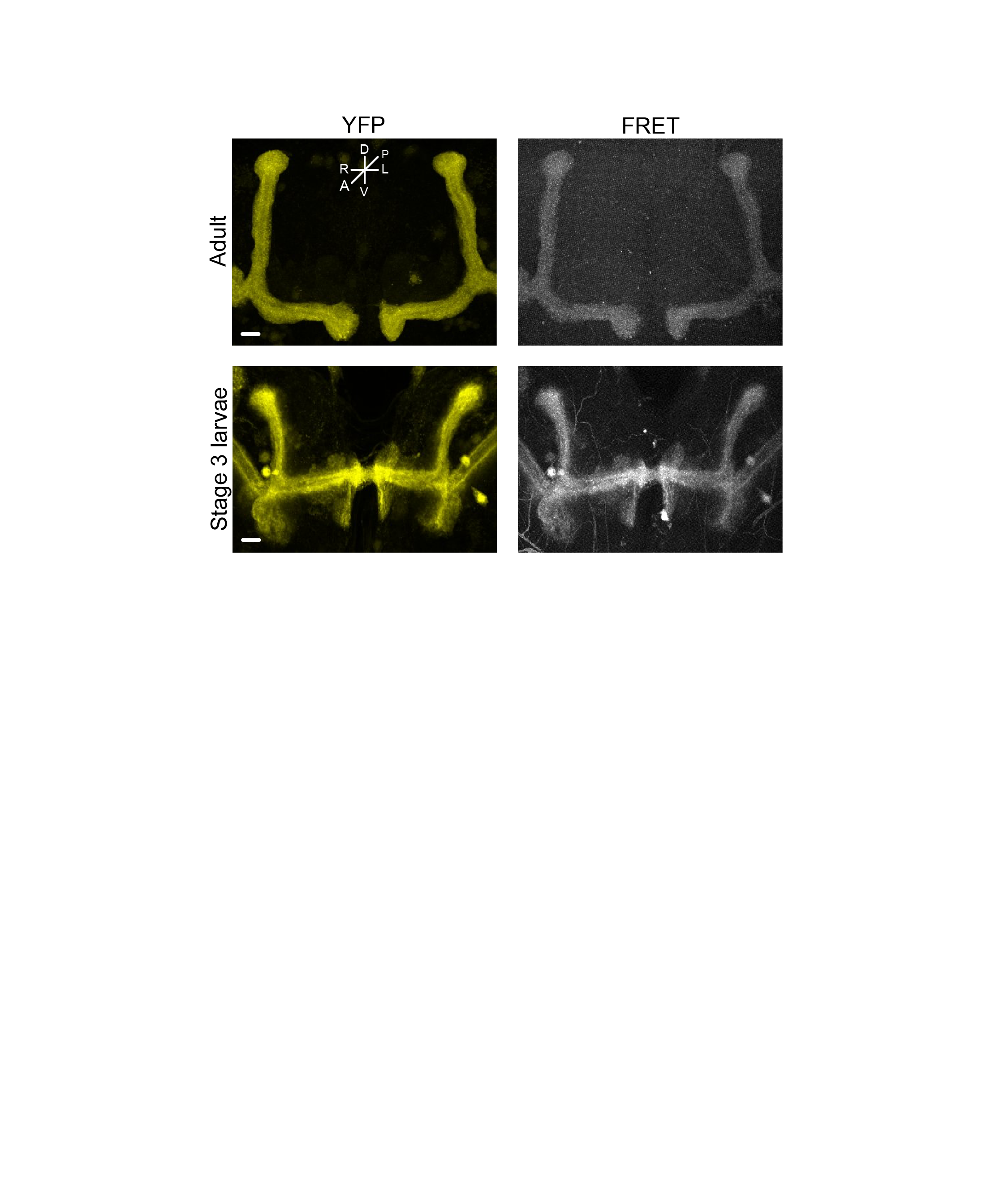

Supplement: S5 Fig — (top) Maximum intensity projection of α and β MB lobes in adult flies. (bottom) Maximum intensity projection of vertical and medial MB lobes in stage 3 larvae. The Abl-FRET biosensor is expressed in the MBs using c739-GAL4 and imaged. Maximum intensity projection of confocal stacks corresponding to YFP and FRET signal are shown. These are presented as examples of the kind of image data that goes into the FRET ratio calculation. Scale bar: 10μm. (TIF) [file pgen.1009287.s005.tif]
